# Quantitative HPLC–UV Study of Lignans in *Anthriscus sylvestris*

**DOI:** 10.3390/molecules27186072

**Published:** 2022-09-17

**Authors:** Dejan Orčić, Sanja Berežni, Neda Mimica-Dukić

**Affiliations:** Faculty of Sciences, University of Novi Sad, 21102 Novi Sad, Serbia

**Keywords:** *Anthriscus sylvestris*, Apiaceae, quantification, validation, HPLC, lignans

## Abstract

Wild chervil (*Anthriscus sylvestris*) is a wild-growing plant from the Apiaceae family, used as a food in Europe and eastern Asia. Due to its high content of lignans known to possess anti-inflammatory, antiproliferative, antiviral and other activities, it represents a potential functional food. However, quantitative data on lignans are still scarce and limited to deoxypodophyllotoxin, nemerosin and yatein. In this paper, a newly developed and validated reverse-phase HPLC–UV method was used to evaluate the content of 14 lignans in both aerial parts and roots of *A. sylvestris*. The most abundant root components were found to be deoxypodophyllotoxin (2.0–42.8 mg/g), nemerosin (2.0–23.4 mg/g), yatein (1.1–18.5 mg/g), podophyllotoxone (0.7–20.5 mg/g), guaiadequiol (0.8–8.3 mg/g) and dimethylmatairesinol (0.1–5.2 mg/g). Despite the high intra-population variability, a general trend of an increased lignan content during plant development could be observed in the root samples, whereas an opposite trend was observed in the herb samples. A validation study indicated that some of the investigated compounds—7-oxoaryltetralins and dibenzylbutyrolactones—have low stability and require cold storage in the dark. Furthermore, dibenzylbutyrolactones were confirmed to undergo a fast *cis*–*trans* isomerization; therefore, only the total content of these isomers should be reported.

## 1. Introduction

*Anthriscus sylvestris* (L.) Hoffm. (Apiaceae), also known as cow parsley or wild chervil, is an herbaceous plant widespread in Europe, North America and Asia. In North America, it has no current use (except for the ornamental “raven’s wing” variety) and is considered a noxious, invasive weed. On the other hand, in Europe and especially in Asia, it is used both as a traditional medicinal plant (as an antitussive, antipyretic, analgesic, diuretic, tonic, digestive, antihypertensive, etc.) and as a food (in soups, salad dressings, etc.) [1,2,3].

The plant is known to be rich in diverse lignans, mainly aryltetralins and dibenzylbutyrolactones [4], which are known to possess anti-inflammatory, antiproliferative, antiviral, antiplatelet aggregation and other activities [5], and thus represents a potential functional food. However, the attempts to measure the exact content of these compounds are scarce and limited to deoxypodophyllotoxin, nemerosin and yatein [1,5,6]. The majority of studies relied upon GC–MS quantification that, while offering a superior resolution, requires sample purification to avoid system contamination and is not adequate for labile compounds (such as unsaturated dibenzylbutyrolactones). Additionally, most studies used only one extraction cycle, which was previously found [7] to result in a severe underestimation of the lignan content (recovery under 70%). All studies confirmed a high variability of the lignan content in both roots and herbs, with deoxypodophyllotoxin reported as low as 0.01 mg/g d.w. in the herb and 0.08 mg/g in the roots and as high as 4.0 mg/g in the herb and 17.3 mg/g in the roots. In addition to seasonal variation [5], even higher intra-population variations were noted [6].

In this paper, we used a newly developed and validated HPLC–UV method to evaluate the content of 14 lignans in the aerial parts and roots of *A. sylvestris* growing on Fruška Gora mountain in Serbia. Additionally, an attempt was made to correlate the chemical composition (the abundance of individual and total lignans) with the plant development phase.

## 2. Results and Discussion

### 2.1. Method Development

To obtain as accurate results as possible, using reasonable resources and time, we employed a previously published method for natural products extraction [7], with some modifications. A solvent volume of 13 mL per 1 g of dried plant material was found to be appropriate to obtain a good compromise between extraction efficiency and extract dilution. Since after 1 h the extraction rate slowed down, the extraction time was shortened to 1 h per cycle. To minimize the risk of degradation, the root extracts were directly analyzed, while the herb extracts (which exhibited lower lignan levels in the preliminary tests) were concentrated fivefold under mild conditions.

**Figure 1:**
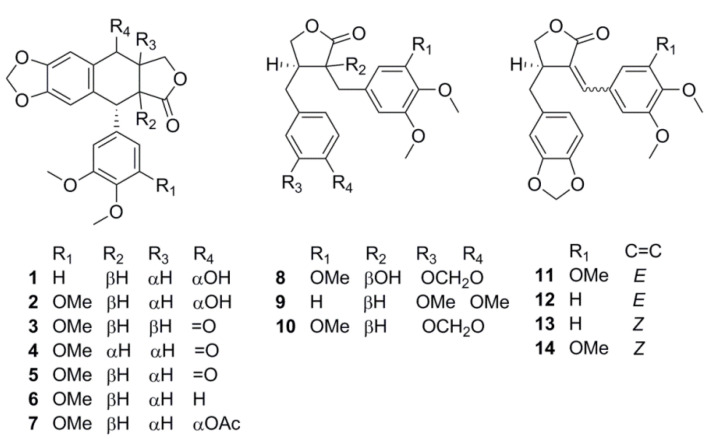
Structures of the investigated compounds.

Based on the prominence in LC–UV–MS chromatograms, 14 lignans (Figure 1) were selected for quantification—aryltetralins 5’-demethoxypodophyllotoxin (**1**), podophyllotoxin (**2**), isopicropodophyllotoxone (**3**), picropodophyllotoxone (**4**), podophyllotoxone (**5**), deoxypodophyllotoxin (**6**), acetylpodophyllotoxin (**7**), dibenzylbutyrolactones guayadequiol (**8**), dimethylmatairesinol (**9**), yatein (**10**), nemerosin (**11**), kaerophyllin (**12**), isokaerophyllin (**13**) and isochaihulactone (**14**). It should be noted that some previously reported [4] minor compounds, such as 5’-demethoxypodophyllotoxone and its stereoisomers, could not be resolved from the more abundant components and were therefore excluded from consideration.

*Anthriscus sylvestris* lignans from the same class (e.g., **1**, **6**, **2** and their esters and stereoisomers) tend to have practically identical UV spectra [4], thus making the identity confirmation problematic in case of additional peaks or shifted peaks. Therefore, mobile phases compatible with both UV/VIS and ESI–MS were chosen, should confirmation by MS become necessary. Since our previously developed HPLC method [4,7] did not exhibit satisfactory performance for quantitative purposes, two different mobile phases (based on 0.1% aq. HCO_2_H, and MeOH or acetonitrile) and two different stationary phases (octadecylsilyl and cyanopropyl) were assessed, using several gradients.

On the octadecylsilyl stationary phase, neither MeOH nor acetonitrile provided the complete separation of all compounds of interest (Figure S1). In fact, unlike MeOH, acetonitrile managed to separate **2** and **6** from minor peaks of **1** and **7**, respectively, but it also caused a complete overlap of the two major components **8** and **5**, a co-elution of **4** with one of its stereoisomers, and an overlap of **12** with **10** or **11**. It was observed that the retention of both hydroxylated lignans (**2** and **8**) was more dependent on the mobile phase composition than the retention of the other lignans. Therefore, various MeOH–acetonitrile mixtures were assessed, and a 35:65 composition was chosen as optimal (Figure S2).

The less common cyanopropyl stationary phase was found to be a promising alternative. MeOH as an eluent exhibited a better performance than the octadecylsilyl phase, facilitating a satisfactory separation of the major peaks, except for **12**, while acetonitrile caused a co-elution of **6** with **9**. Both mobile phases allowed a poor separation of unsaturated dibenzylbutyrolactones. Therefore, the octadecylsilyl phase was chosen for subsequent work.

The majority of compounds were monitored at 280 nm. An additional wavelength of 330 nm was used for compounds with extended delocalization, i.e., 7-oxoaryltetralins (**3**–**5**) and unsaturated dibenzylbutyrolactones (**11**–**14**), since it offered similar sensitivity but better selectivity.

### 2.2. Method Validation

#### 2.2.1. Chromatographic Performance

Chromatographic performance data are presented in Table S1. The retention times (*t*_R_) showed excellent repeatability, with standard deviations (as determined from real samples) ranging from 0.011 to 0.032 min (0.25–0.63%) for the root extracts and from 0.009 to 0.038 min (0.19–1.12%) for the herb extracts. For all the investigated compounds, the retention factor *k* ranged from 4.4 (**1**) to 11.6 (**14**), which is acceptable and satisfies the requirements [8]. For the standards, the peaks were generally well resolved from their neighbors, with *R*s typically exceeding 3 (vs. ≥1.5, as required by AOAC [9]), and exhibited satisfactory symmetry. The resolution for the real samples was somewhat worse, due to their complexity (it was previously established [4] that at least 46 lignans are present in *A. sylvestris*), but generally, at least a baseline separation was achieved. The most important exception was **12**, which could not be separated from **10** under the studied conditions. In this case, it was possible to selectively detect **12** at 330 nm, while the peak area of **10** had to be corrected considering the contribution of **12** (found to be *A*_KAE,280_ = 0.530 *A*_KAE,330_) before reading off a calibration curve.

#### 2.2.2. Identity

The retention times of the analytes in the extracts were well within the tolerance of ± 0.1 min of the *t*_R_ for the calibration standards prescribed by SANTE [10] and of ±2.5% indicated by the European Commission [8]. The absorption spectra of analytes and standards visually matched, and the absorption maxima were within the DAD resolution limit of 2 nm [8]. The sole exception was the overlapped peak of **10** and **12**, which featured the maxima for both compounds. The identity of the peaks was further confirmed by co-chromatography, with the samples used in the trueness study. The peak width at half-maximum for the spiked samples was between 91% and 110% of the peak width for the non-spiked samples [8], except for **1** and **14** for the herb extracts (136% and 112%, respectively) and **6** for the root extracts (82%). Finally, the selected samples were analyzed by LC–MS/MS (Figure S3), and the peaks’ identities were confirmed by comparison of the MS^2^ spectra with those in the in-house created spectral library [4].

#### 2.2.3. Linearity

The previous results indicated a high variability of the samples’ composition, stemming from both environmental factors and plant development phase. To accommodate for this, a calibration study covered a wide concentration range of standards prepared in 80% aqueous methanol (Table S2), from as low as 0.125 µg/mL to as high as 1000 µg/mL (at 8–10 levels), with the concentration range for each compound adapted to the highest expected contents. The calibration curves exhibited satisfactory linearity over the investigated concentration range, with *r*^2^ ≥ 0.999. Several compounds, especially **1** and **4**, exhibited a somewhat better agreement with a quadratic curve when the full concentration range was used. In all cases, the calibration curve intercept was negligible (lower than twice the standard deviation of the intercept) or close to negligible (in the case of **9**).

#### 2.2.4. Detection and Quantitation Limits

LoD and LoQ were determined by an ICH approach [11], based on the residuals’ standard deviation for the calibration curve. To avoid any potential heteroscedasticity problems, only the lowest five levels of the calibration curves were used. The LoQ values varied in the range of 0.1–6.6 µg/mL for the vial, translating to 0.006–0.37 mg/g for root and 0.0011–0.073 mg/g for herb material (Table S2). In almost all cases, the amounts of lignans exceeded the LoQ.

#### 2.2.5. Trueness and Precision

Due to the unavailability of certified reference materials, trueness was determined by a spiking study, using samples poor in lignans as a matrix. Spiking was performed in four replicates. The obtained recovery values were within the 76.5–106.9% range (Table S3), which is satisfactory. Recovery of **5** from the herb was found to be less efficient, only 63.0%. This was likely due to its instability (see Section 2.2.7), since the total recovery of **5** and its main degradant, **4**, was 73.1% and 78.1% in herb and root, respectively.

Precision (repeatability) was determined from the samples used for the trueness study. Due to the low concentration in non-spiked samples, the spiked samples were used for the calculation. All compounds exhibited excellent precision, with relative standard deviations <10%, which is well within the criteria of ≤15% established by the FDA [12] and ≤20% established by SANTE [10].

#### 2.2.6. Robustness

The robustness of the extraction step was studied by varying the extraction time per step (60 ± 15 min) and the extraction solvent (80 ± 10% MeOH). For the herb samples, 80% MeOH was consistently the best solvent, providing the highest yield (Figure S4). The absolute change of ±10% in MeOH content resulted in up to a 48% change in yield. For the majority of the compounds, root samples’ extraction was less affected by the solvent composition, with deviations mostly within the margins of error (as determined by the precision study). In most cases, 80% MeOH provided a slightly lower, but still acceptable yield. For both herb and root samples, to keep the yield within ±5% of the nominal value, the extraction solvent should contain 80 ± 3% MeOH.

The extraction cycles of 60 min consistently provided the highest yield (Figure S5). Surprisingly, prolonging the extraction time actually resulted in a decreased lignans content. To keep the yield within ±5% of the nominal value, the extraction time should be kept within ±4 min for the herb samples and within +4/–10 min for the root samples.

A decrease of the methanol content in the mobile phase by 5% (absolute) decreased the retention times by up to 2% (Figure S6). On the other hand, an increase by 5% significantly increased the retention times; hence, the mobile phase composition tolerance was 34 ± 4% of MeOH.

#### 2.2.7. Stability

The *cis*–*trans* isomerization of unsaturated dibenzylbutyrolactones, catalyzed by factors such as light and acids, has been long known [4,13,14]. In our study, it was observed that the initially pure standards of **11** and **13**, two years old, could be used for the calibration of the other isomers as well, with the reasonable assumption that the peak area ratio of the two isomers is proportional to the amount ratio. However, during the calibration study, it was also observed that the peak area ratios varied between the calibration levels. A study with the standards of **13** injected in random order demonstrated that the isomers ratio was affected both by the passage of time (with the **12**/**13** ratio increasing) and, surprisingly, by their total concentration (with the **12**/**13** ratio decreasing). Since this is also likely to occur in the samples during the analysis, the observed isomeric ratio cannot be considered representative of the plant composition, and the results should only be reported as a sum of *cis* and *trans* isomers.

To evaluate the sample stability during storage, the aliquots of representative root and herb extracts were kept for one month at room temperature in the dark, at room temperature under normal lab lighting (daylight and fluorescent illumination), at 4 °C, −20 °C and −80 °C, the last temperature being used as a control.

By the second week of storage, a significant conversion of **5** into **4** was already observed, even at 4 °C in the dark (to 80% and 56% of the initial amount in root and herb extracts, respectively). The effect was more pronounced at room temperature, with complete or near-complete conversion. Additionally, in the illuminated root extracts, but not in the ones kept in the dark, significant isomerization of **12** into **13** and of **11** into **14** was observed, resulting in the increase of the *cis*-isomer content by 4.6 and 5.0 times, respectively. Interestingly, the isomerization was minimal in the herb extracts, likely due to the photoprotective effects of plant pigments.

By the fourth week of storage, **5** was degraded to 69.4% in the root and to 42% in the herb extracts at 4 °C and completely at room temperature (Figure S7). The main conversion products were found to be **4** and an unknown compound that possessed a molecular weight of 412 but exhibited a UV spectrum different from those of the other 7-oxoaryltetralins and, thus, was likely not the fourth isomer, i.e., isopodophyllotoxone. The peak area of **4** significantly increased even in the samples held at −20 °C. The peaks of **13** and **14** were increased in the root extract by 6.2 and 7.0 times, respectively, upon storage at room temperature under illumination. Since the photoprotective pigments were also significantly degraded after four weeks, as evidenced by the change in color, the amount of **13** and **14** was also increased in the herb samples, by 2.4 and 1.4 times, respectively.

To summarize, while some compounds appeared to be sufficiently stable (**6**, **8**, **9**, **10**) or unstable but within acceptable limits of ±15% (**2**, **11**), even at room temperature, others (7-oxoaryltetralins and *cis*-unsaturated dibenzylbutyrolactones) changed significantly. In addition to a decrease in the peak area of the unstable compounds, their degradation could also impede the analysis of other analytes. For example, it was found that the peak of **3** overlapped with that of a degradation artifact of **5**, tentatively identified as a dihydroxylated derivative of either **5** or its stereoisomers. The results indicate that the *A. sylvestris* extracts should be stored in a deep freeze (for up to four weeks) or a freezer (up to two weeks) and protected from light exposure.

### 2.3. Samples Analysis

**Figure 2:**
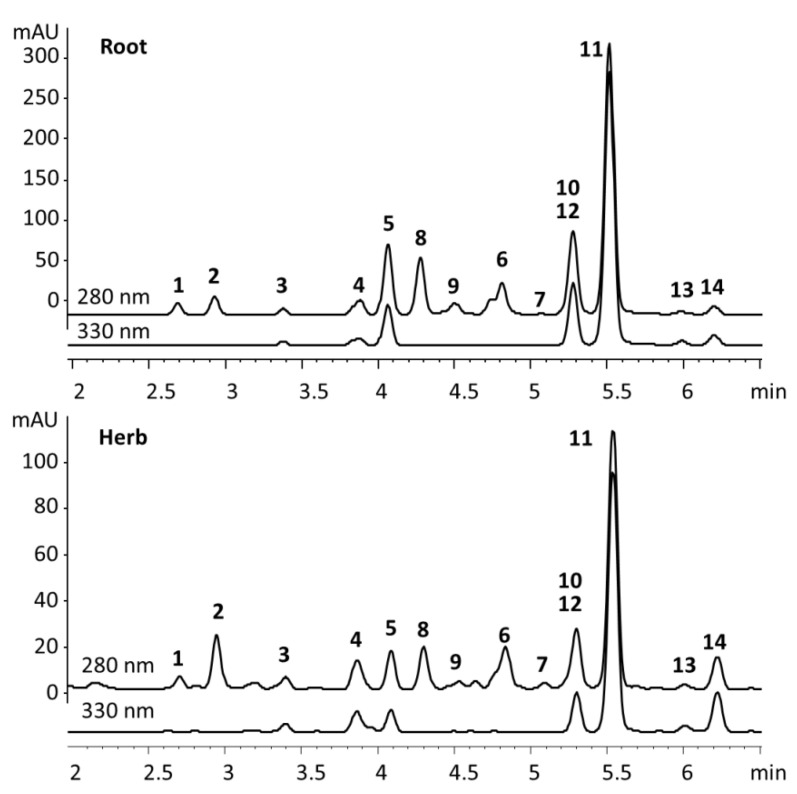
UV chromatograms of representative root and herb extracts.

The lignan profile of the root samples was dominated (78.9–91.7% of the total lignans) by methylenedioxy,trimethoxy-substituted aryltetralins, dibenzylbutyrolactones, and their oxygenated derivatives (Figure 2, Table 1). In the majority of the samples, the dominant lignan was **6**, as previously found [6,15]. As previously reported, the concentration varied widely, from 2.0 mg/g to 42.8 mg/g (17.3–55.6% of the total lignans), with the median value of 13.8 mg/g. Unlike a previous study that found only 0.25–0.76 mg/g of **10** [6], in several samples its content exceeded that of **6** and reached up to 18.5 mg/g. Another abundant component, a mixture of anhydropodorhizol (*Z*)- and (*E*)-isomers (**11** + **14**), amounted up to 2.1–24.2 mg/g, also exceeding the previously published values (0.49–0.73 mg/g), but only in one sample rising above the content of **6**. Another prominent lignan, neglected in previous quantitative studies, **5**, amounted up to 20.5 mg/g and in some cases actually exceeded the content of **6**. The total lignan content, calculated as a sum of the contents of the individual determined components, ranged from 11.3 mg/g to 106.2 mg/g (median 40.0 mg/g) and was in all but two samples higher than previously reported [15]. Despite the high intra-population variability, a general trend of an increase in content during plant development could be observed, both for the total lignans and for the majority of individual compounds (Figure S8). This behavior is opposite to that previously reported [5].

The total lignan content in the herb samples was several times lower than in the roots, ranging from 2.1 to 15.1 mg/g (median 5.9 mg/g). Compounded with an inherently more complex matrix, due to an active metabolism, this makes the herbs a less suitable source for the isolation of lignans, but still a viable component of functional foods. It should be noted that previous studies indicated that the lignan content in the aerial parts of indoor-cultivated *A. sylvestris* can be close to or even higher than that in roots, in contrast to what observed for wild-growing specimens [6]. The lignan profile of the aerial parts was characterized by higher percentages of aryltetralins **6** (that was consistently the most abundant lignan), **2** and **7**, and lower contributions of dibenzylbutyrolactones **10** (that is a biosynthetic precursor of aryltetralins), **8** and **11**. As for the roots, the high intra-population variability hindered discerning trends. However, the median values generally decreased during plant development, which is consistent with previous findings [5].

The reasons for a high intra-population variability are currently unknown. It is believed that lignans have protective (insecticidal, antifungal, etc.) functions in plants, but attempts to elicit their production by stressors failed [16]. Thus, lignans are not phytoalexins but phytoanticipins, and the genetic differences between specimens, rather than the differences in endured stress, are probably the cause of variations [6]. A high variability also implies that there is no significant selection pressure for phenotypes with a higher lignan content.

## 3. Materials and Methods

### 3.1. Samples and Reagents

The roots and herbs of *Anthriscus sylvestris* were collected from Fruška Gora mountain (45.15727° N/19.79375° E) in Serbia, on 17.4.2021 (vegetation phase), 27.4.2021 (flowering), 17.5.2021 (flowering and fruit-bearing) and 4.6.2021 (fruit-bearing phase). Five to eight specimens were sampled each time. The voucher specimens were confirmed and deposited at the Herbarium of the Department of Biology and Ecology (BUNS Herbarium), Faculty of Sciences, University of Novi Sad, under the designations 2-2249 to 2-2252. The material was chopped, air-dried at room temperature and pulverized.

Methanol was purchased from Sigma-Aldrich (Steinheim, Germany), acetonitrile from Honeywell (Seelze, Germany), and formic acid from Merck (Darmstadt, Germany). The standards of 5’-demethoxypodophyllotoxin, podophyllotoxin, isopicropodophyllotoxone, picropodophyllotoxone, podophyllotoxone, deoxypodophyllotoxin, acetylpodophyllotoxin, guayadequiol, dimethylmatairesinol, yatein, nemerosin, isochaihulactone, kaerophyllin and isokaerophyllin were isolated in our lab from *A. sylvestris* roots, as previously described [4].

### 3.2. Extraction

Accurately weighted samples (180 mg) were extracted by maceration with 80% aq. MeOH (2.4 mL per cycle, 4 cycles of 1 h each) with constant shaking. The extracts were pooled and diluted to 10.0 mL. Aliquots of the herb extracts (5.0 mL) were subsequently evaporated at 35 °C under reduced pressure and re-dissolved (1.0 mL). All extracts were held in the dark at 4 °C until the HPLC analysis.

### 3.3. HPLC analysis

The extracts were analyzed on an Agilent Technologies series 1100 HPLC consisting of a vacuum degasser, a binary pump, a manual injector, a thermostated column compartment and a diode-array detector. The samples (5 μL) were injected onto a Zorbax Eclipse XDB-C18 (1.8 μm, 4.6 × 50 mm) column held at 50 °C. The components were eluted by a mobile phase consisting of 0.1% aq. HCO_2_H (phase A) and 35% MeOH in acetonitrile (phase B), delivered at 1 mL/min in gradient mode: 0 min 35% B, 14–16 min 100% B, post-time 3 min. The signals at 220 nm, 280 nm and 330 nm (16 nm bandwidth) were monitored, with 550 nm (100 nm bandwidth) used as a reference, together with a full scan in the 200–400 nm range. The results were processed in ChemStation for LC 3D Systems software, rev. B.02.01-SR2(260). The compounds were quantified based on the external standard (ESTD) method.

### 3.4. HPLC–MS/MS Analysis

The extracts were analyzed on an Agilent Technologies series 1200 HPLC which was further coupled with an Agilent Technologies series 6410 electrospray ionization triple-quad mass spectrometer. The HPLC parameters were the same as described in Section 3.3. The effluent was forwarded into the ion source, with a nebulizer gas (N_2_) pressure of 50 psi, a drying gas (N_2_) flow of 10 L/min, a temperature of 350 °C, a capillary voltage of 4000 V, a fragmentor voltage of 135 V, and positive ion mode. The data were acquired in MS2Scan mode (*m*/*z* range 100–1000) and Product Ion Scan mode (with [M+H]^+^ ions as precursors and collision energy of 10 V and 30 V). The results were processed in Agilent MassHunter software (ver. B.06) coupled with NIST MS Search (ver. 2.0) with an in-house created spectral library [4].

## 4. Conclusions

An HPLC–UV method, using an octadecylsilyl stationary phase and an acidified H_2_O–acetonitrile–MeOH mobile phase, was successfully employed to quantify 14 common aryltetralin and dibenzylbutyrolactone lignans within 7 min. The developed method exhibited satisfactory analytical performances. A total of 29 root and 27 herb samples of *Anthriscus sylvestris* from Fruška Gora mountain, Serbia, were studied. With a total lignan content of 1.1–11% (d.w.), *A. sylvestris* root was proven to be a rich source of these highly bioactive compounds, while the aerial parts exhibited a significantly lower, but still high, total lignans content of 0.2–1.5%, d.w. Hence, this root vegetable represents a promising component of functional foods.

## Figures and Tables

**Table 1:** Content of lignans in root (R) and herb (H) extracts of *A. sylvestris*, in mg/g d.w. The results are presented as the average ± standard deviation of 5–8 biological replicates.

Cpd	R(17.4.2021.)	R(27.4.2021.)	R(17.5.2021.)	R(4.6.2021.)	H(17.4.2021.)	H(27.4.2021.)	H(17.5.2021.)	H(4.6.2021.)
**1**	0.32 ± 0.21	0.35 ± 0.18	0.49 ± 0.70	0.72 ± 0.45	0.078 ± 0.060	0.069 ± 0.048	0.042 ± 0.025	0.037 ± 0.026
**2**	1.9 ± 1.2	2.22 ± 0.69	3.5 ± 3.3	3.6 ± 2.5	0.79 ± 0.27	0.95 ± 0.28	0.82 ± 0.16	0.45 ± 0.20
**3**	0.038 ± 0.018	0.047 ± 0.033	0.058 ± 0.022	0.089 ± 0.046	0.020 ± 0.011	0.026 ± 0.023	0.0127 ± 0.0032	0.0095 ± 0.0054
**4**	0.19 ± 0.11	0.31 ± 0.12	0.43 ± 0.21	0.71 ± 0.39	0.105 ± 0.089	0.125 ± 0.081	0.069 ± 0.034	0.085 ± 0.073
**5**	3.8 ± 2.1	5.1 ± 2.1	7.3 ± 4.3	11.6 ± 4.8	0.52 ± 0.48	0.40 ± 0.13	0.307 ± 0.090	0.23 ± 0.14
**6**	11.0 ± 8.4	11.0 ± 4.2	18.6 ± 5.5	22 ± 13	4.3 ± 1.9	2.28 ± 0.63	3.05 ± 0.90	2.23 ± 0.90
**7**	0.15 ± 0.12	0.42 ± 0.21	1.18 ± 0.85	0.58 ± 0.68	0.22 ± 0.26	0.155 ± 0.088	0.190 ± 0.062	0.091 ± 0.033
**8**	2.14 ± 0.62	2.8 ± 1.5	3.9 ± 2.1	3.6 ± 1.8	0.37 ± 0.24	0.32 ± 0.20	0.215 ± 0.068	0.090 ± 0.080
**9**	1.43 ± 0.53	1.42 ± 0.76	2.3 ± 1.0	2.3 ± 1.6	0.22 ± 0.14	0.151 ± 0.091	0.113 ± 0.078	0.067 ± 0.067
**10**	3.7 ± 2.1	6.0 ± 1.7	9.6 ± 4.9	9.0 ± 3.3	0.80 ± 0.64	0.76 ± 0.80	0.63 ± 0.23	0.34 ± 0.16
**11+14**	5.8 ± 1.6	5.9 ± 3.7	8.4 ± 3.3	10.1 ± 7.1	1.00 ± 0.35	0.94 ± 0.36	0.77 ± 0.23	0.58 ± 0.39
**12+13**	0.96 ± 0.38	1.04 ± 0.46	1.11 ± 0.64	2.0 ± 1.1	0.24 ± 0.22	0.17 ± 0.10	0.085 ± 0.025	0.086 ± 0.059
Σ	31 ± 12	37 ± 13	57 ± 23	66 ± 26	8.6 ± 3.9	6.3 ± 2.5	6.3 ± 1.3	4.3 ± 1.9

## Data Availability

The data presented in this study are available within the article and the Supplementary Material. Raw datafiles are available on request from the corresponding author (D.O.).

## Supplementary Materials

The following supporting information can be downloaded at: https://www.mdpi.com/article/10.3390/molecules27186072/s1, Figure S1. Typical chromatograms obtained by methanol- and acetonitrile-based mobile phases and octadecylsilyl (Zorbax Eclipse XDB-C18, 1.8 μm, 4.6 × 50 mm) and cyanopropyl-based stationary phases (Zorbax SB-CN, 1.8 μm, 4.6 × 50 mm), Figure S2. Compounds’ retention on an octadecylsilyl stationary phase as a function of the acetonitrile content in a mixture with methanol, Figure S3. ESI(+)-MS^2^ spectra of the investigated compounds, at collision energies of 10–40 V, Figure S4. The effect of the extraction solvent composition (70%, 80% and 90% MeOH) on the extraction yield (shown as a percentage of the yield with 80% MeOH) from the aerial parts and roots, Figure S5. The effect of the extraction time (45 min, 60 min, 75 min) on the extraction yield (shown as a percentage of the yield at 60 min) from the aerial parts and roots, Figure S6. The effect of the MeOH content in the mobile phase (30%, 35%, 40%) on the retention time (shown as the relative retention time, RRT, against the retention time obtained with 35% MeOH), Figure S7. Stability evaluation after four weeks of storage: 280 nm chromatograms of the samples stored at −80 °C, −20 °C, 4 °C, and room temperature in the dark and under normal illumination, Figure S8. Changes of the lignan content during plant development, Table S1. Chromatographic performance parameters, Table S2. Linearity and detection ability parameters, Table S3. Trueness and precision parameters.
