# Supplementary material for: Quantitative HPLC–UV Study of Lignans in Anthriscus sylvestris
Source: Molecules. 2022 Sep 17;27(18):6072. doi: 10.3390/molecules27186072 (PMC9500830; doi:10.3390/molecules27186072)
Supplement: Supplementary file 1 [file molecules-27-06072-s001.zip › molecules-1912934-supplementary.pdf]

Supporting Information

Quantitative HPLC-UV study of lignans in *Anthriscus sylvestris*

Dejan Orčić [1,*], Sanja Berežni [1] and Neda Mimica-Dukić [1,*]

[1] University of Novi Sad, Faculty of Sciences, 21000 Novi Sad, Serbia
* Correspondence: neda.mimica-dukic@dh.uns.ac.rs (N.M-D.); dejan.orcic@dh.uns.ac.rs (D.O.); Tel.: +381214852757 (N.M.-D.); +381214852765 (D.O)

Table of contents

Figure S1. Typical chromatograms obtained by methanol- and acetonitrile-based mobile phase, and octadecylsilyl (Zorbax Eclipse XDB-C18, 1.8 µm, 4.6 x 50 mm) and cyanopropyl-based stationary phase (Zorbax SB-CN, 1.8 µm, 4.6 x 50 mm). 3

Figure S2. Compounds' retention on an octadecylsilyl stationary phase as a function of acetonitrile content in mixture with methanol. 3

Table S1. Chromatographic performance parameters. 4

Figure S3. ESI(+)-MS^2 spectra of the investigated compounds, at collision energies 10–40 V. 5

Table S2. Linearity and detection ability parameters. 19

Table S3. Trueness and precision parameters. 19

Figure S4. The effect of the extraction solvent composition (70 %, 80 % and 90 % MeOH) on the extraction yield (given as percentage of yield with 80 % MeOH) from aerial parts (top) and roots (bottom). 20

Figure S5. The effect of the extraction time (45 min, 60 min, 75 min) on the extraction yield (given as percentage of yield at 60 min) from aerial parts (top) and roots (bottom). 21

Figure S6. The effect of MeOH content in the mobile phase (30 %, 35 %, 40 %) on the retention time (given as relative retention times, RRT, against the retention times obtained with 35 % MeOH). 22

Figure S7. Stability evaluation after four weeks of storage: 280 nm chromatograms of sample stored at –80 °C (blue) and at –20 °C, 4 °C, and room temperature in dark and under normal illumination (red). 23

Figure S8. Changes of lignans content during the plant development. 24

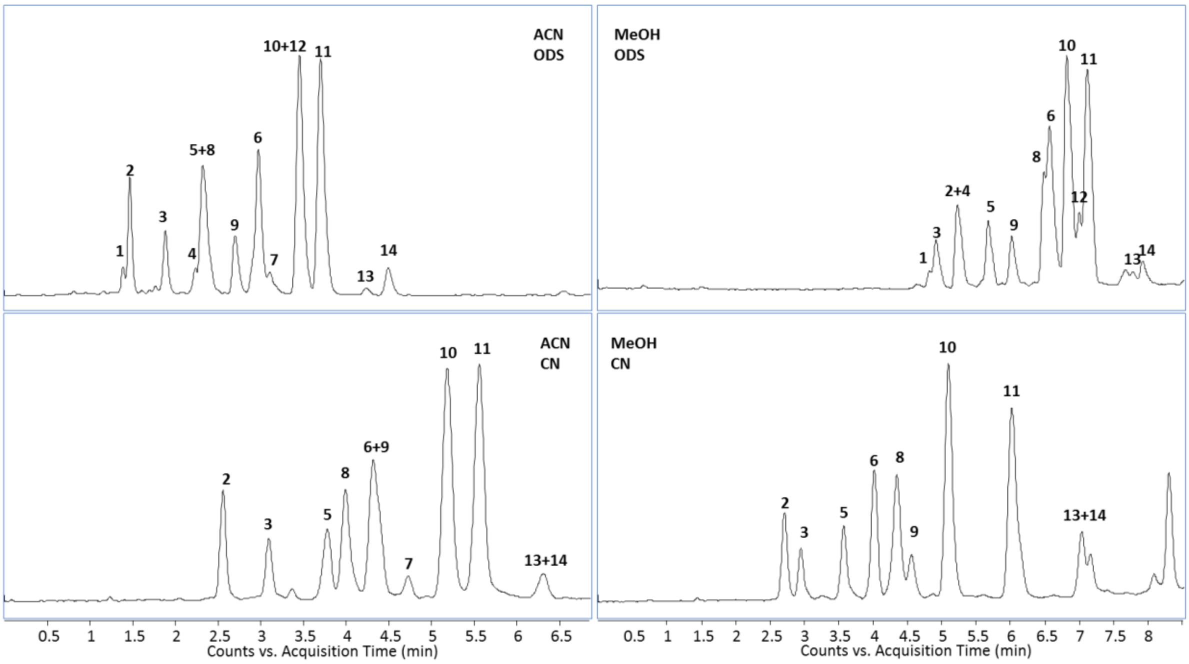

Figure S1. Typical chromatograms obtained by methanol- and acetonitrile-based mobile phase, and octadecylsilyl (Zorbax Eclipse XDB-C18, 1.8 µm, 4.6 x 50 mm) and cyanopropyl-based stationary phase (Zorbax SB-CN, 1.8 µm, 4.6 x 50 mm).

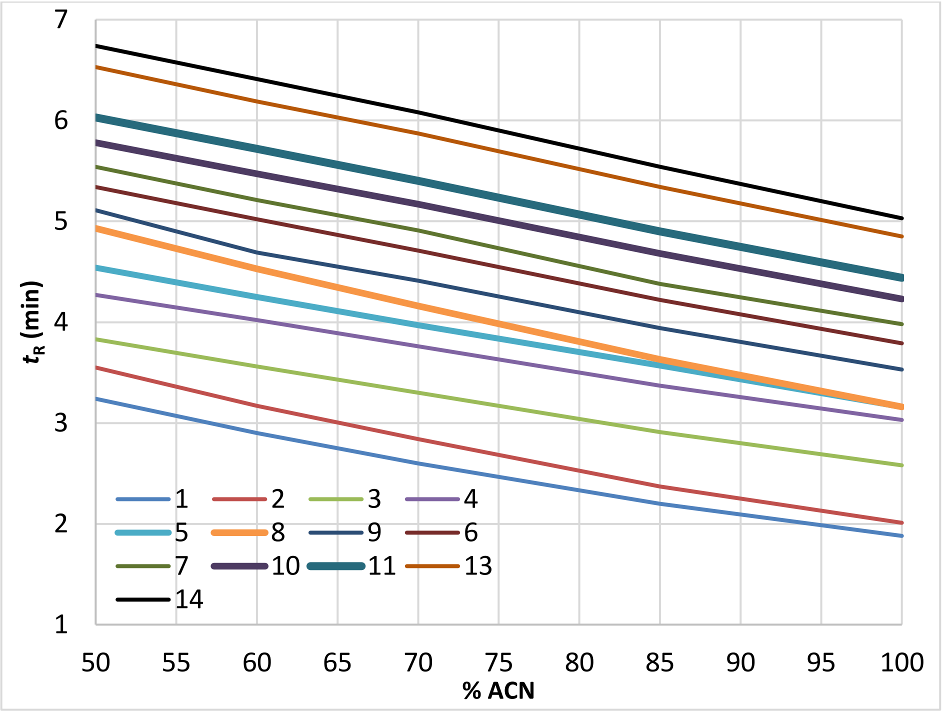

Figure S2. Compounds' retention on an octadecylsilyl stationary phase as a function of acetonitrile content in mixture with methanol.

Table S1. Chromatographic performance parameters.

	standard		root			herb		
Cpd	t_R/min	*k*	t_R/min	RSD(t_R)/%	*Rs*[a]	t_R/min	RSD(t_R)/%	*Rs*[a]
**1**	2.68	4.4	2.69	0.40	1.8	2.67	0.34	2.3
**2**	2.92	4.9	2.93	0.39	2.1	2.91	0.32	2.3
**3**	3.39	5.8	3.38	0.35	1.9	3.40	1.12	1.3
**4**	3.85	6.8	3.87	0.41	1.5	3.83	0.26	2.0
**5**	4.05	7.2	4.06	0.34	1.5	4.05	0.26	2.0
**6**	4.80	8.8	4.81	0.30	1.5	4.79	0.23	1.5
**7**	5.05	9.2	5.04	0.63	1.5	5.05	0.23	1.5
**8**	4.27	7.7	4.28	0.32	1.5	4.26	0.26	1.7
**9**	4.49	8.1	4.50	0.33	1.5	4.49	0.26	1.7
**10**	5.26	9.7	5.27	0.27	[b]	5.26	0.21	[b]
**11**	5.50	10.2	5.51	0.27	2.1	5.50	0.20	1.6
**12**	5.25	9.7	5.27	0.28	2.1	5.26	0.50	2.1
**13**	5.97	11.2	5.98	0.34	1.4	5.97	0.21	1.7
**14**	6.19	11.6	6.19	0.25	1.4	6.18	0.19	0.7

[a] spiked sample, from trueness study

[b] complete overlap with **12**

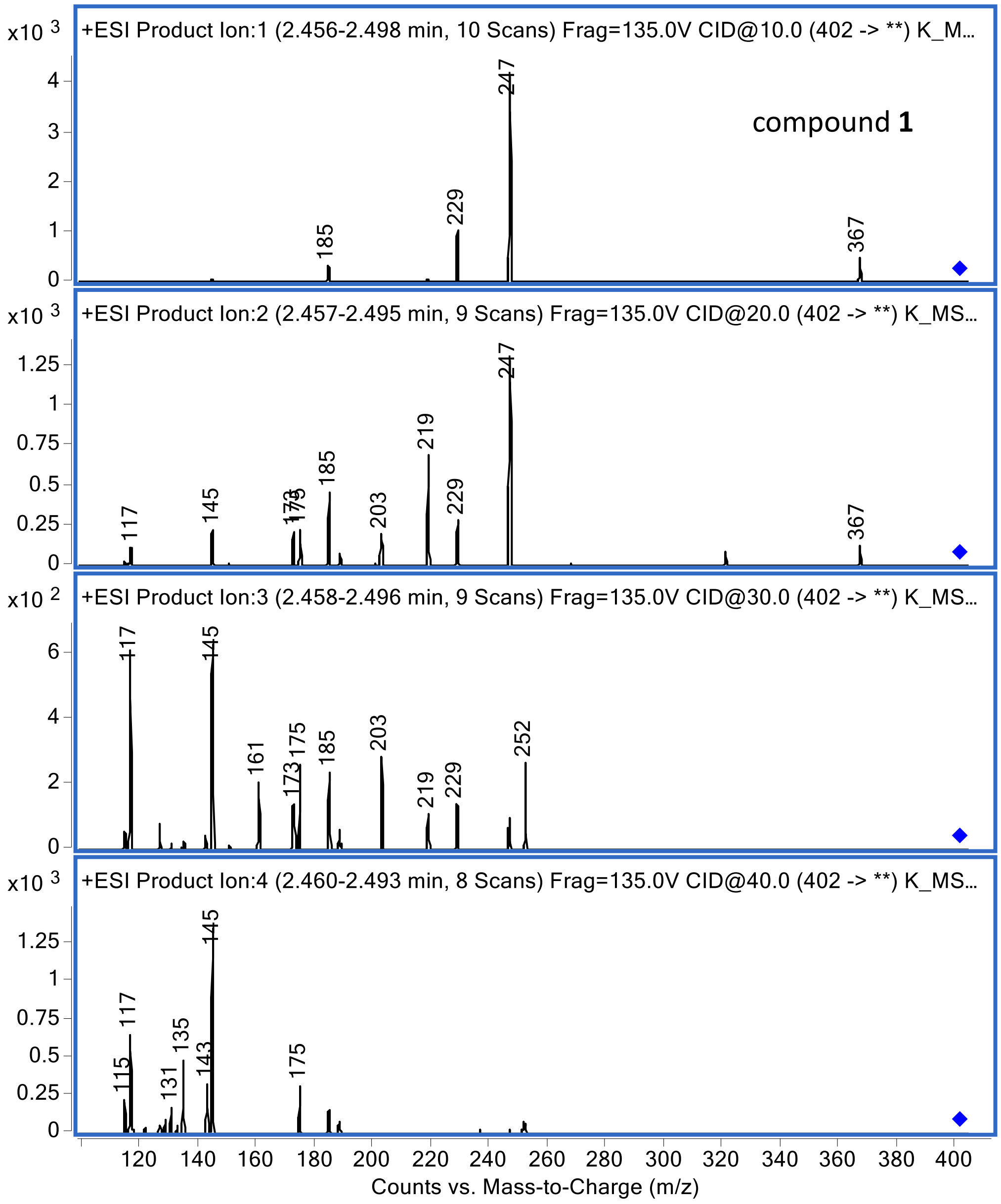

Figure S3. ESI(+)-MS^2 spectra of the investigated compounds, at collision energies 10–40 V.

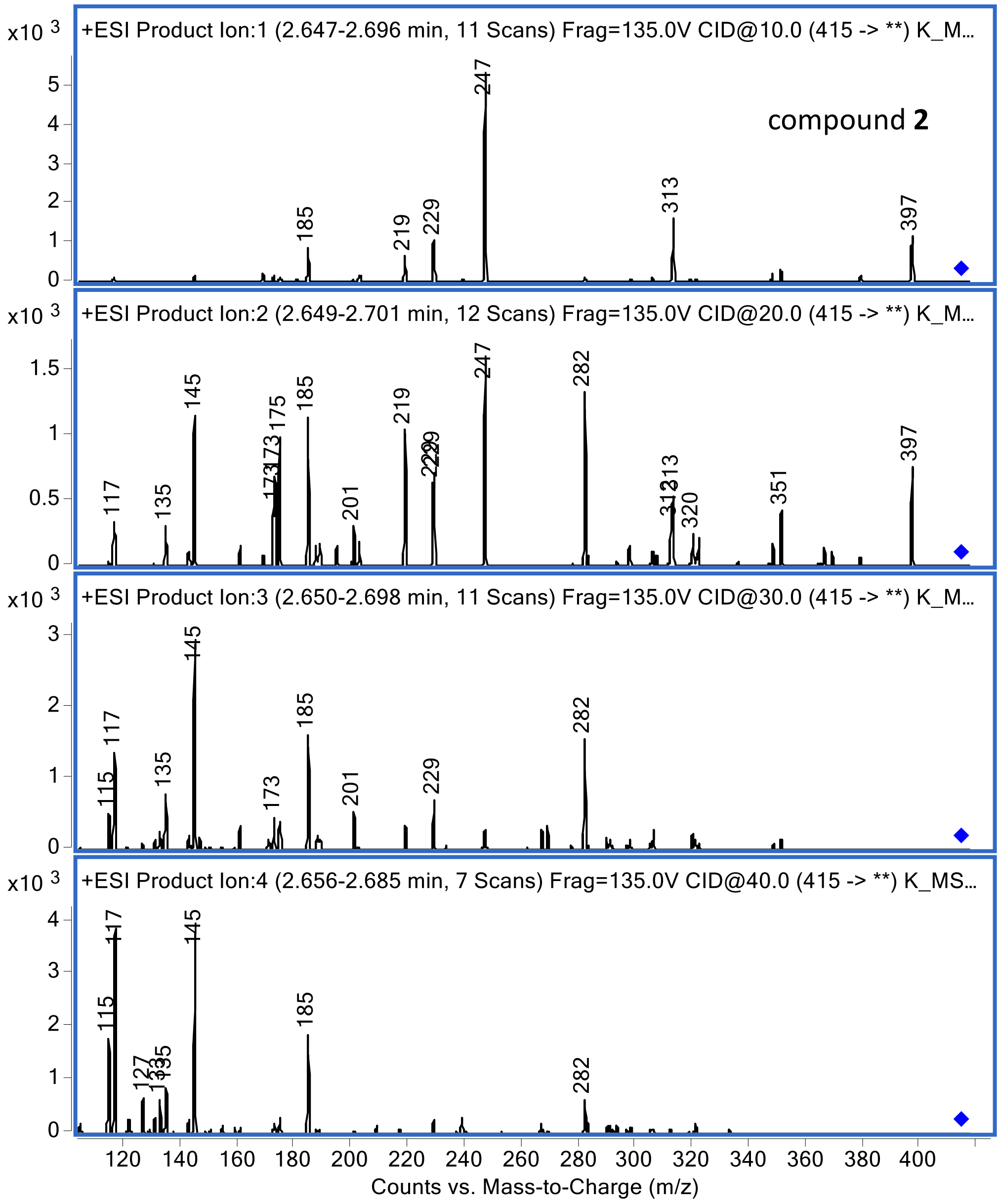

Figure S3. (continued)

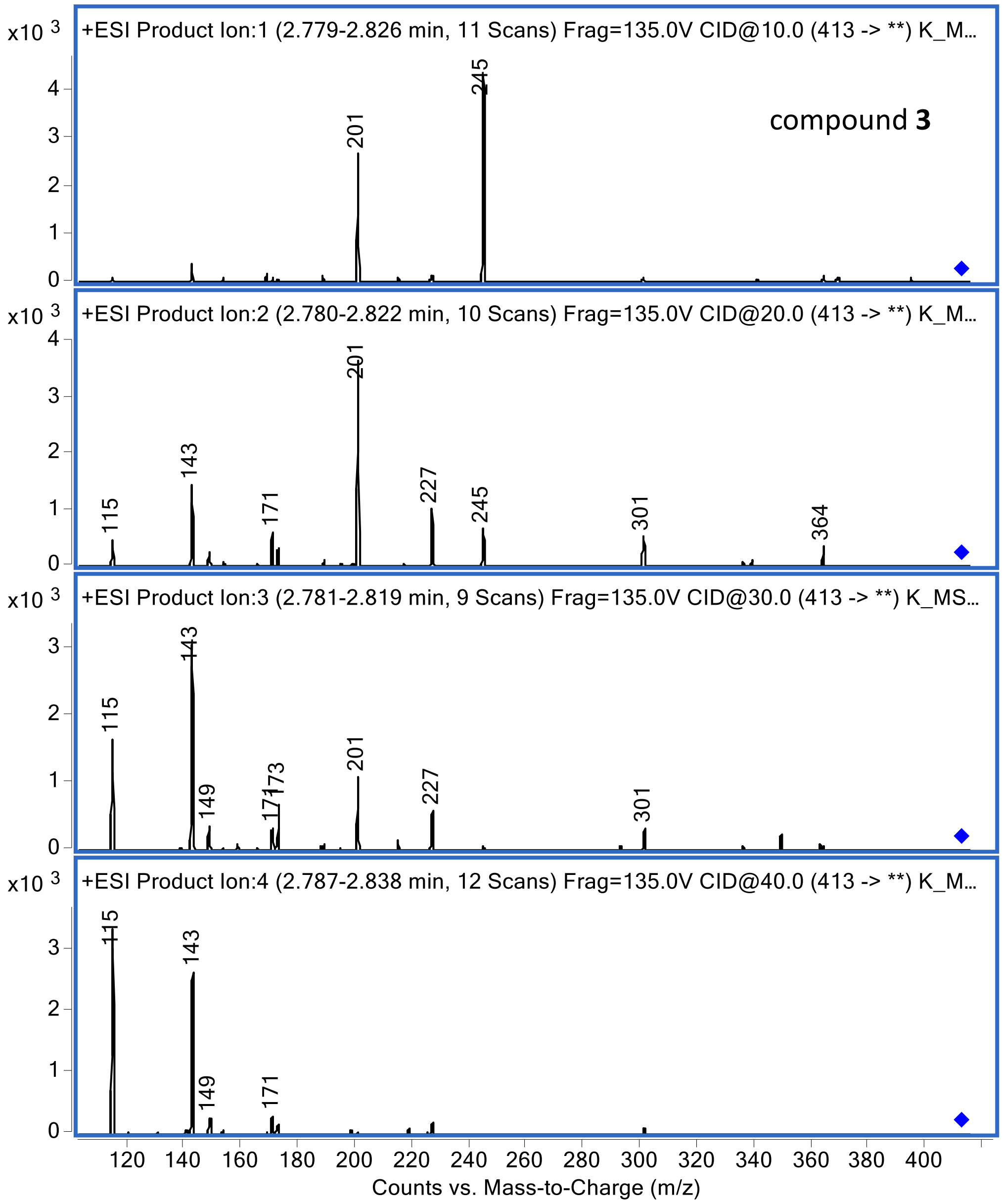

Figure S3. (continued)

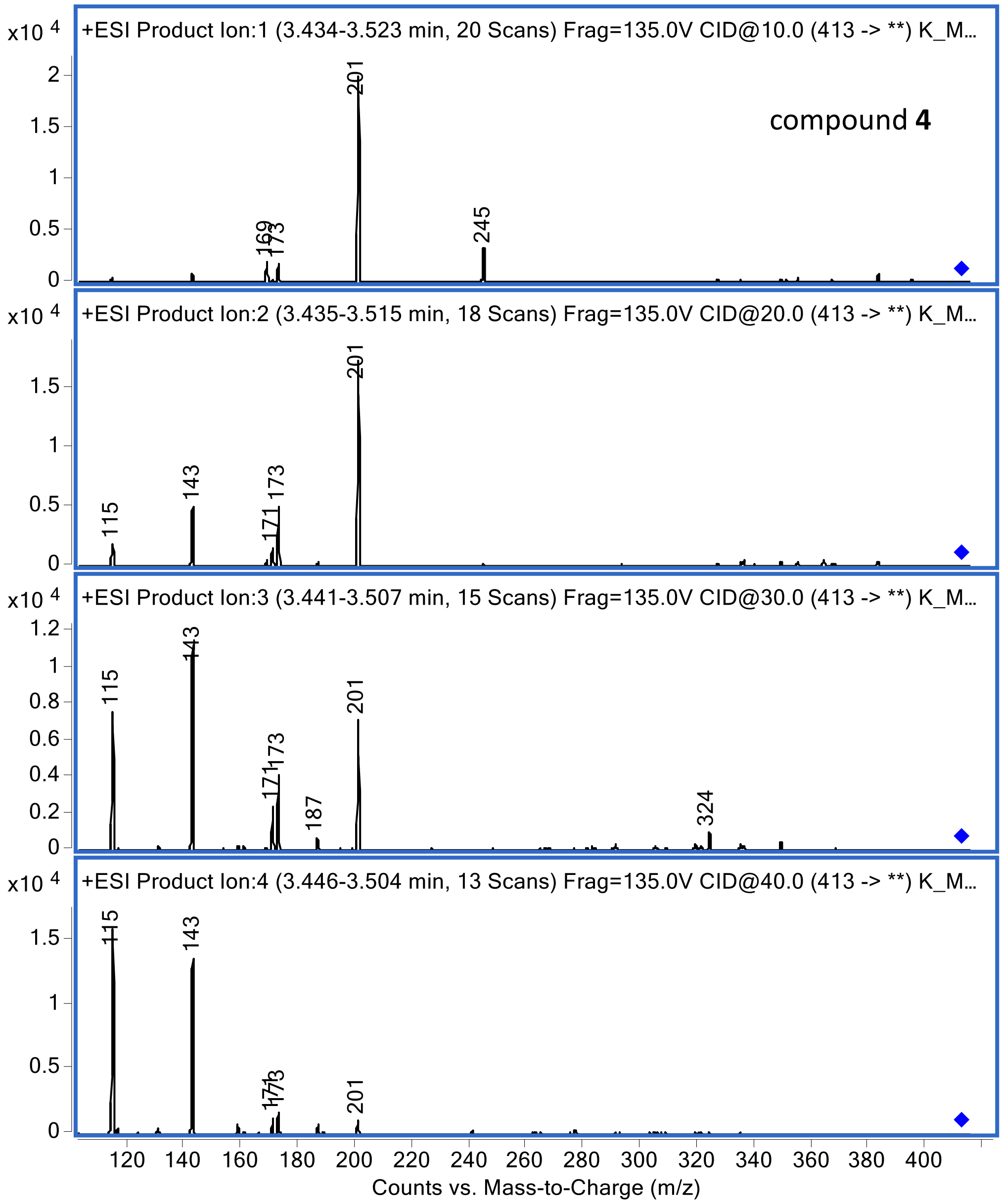

Figure S3. (continued)

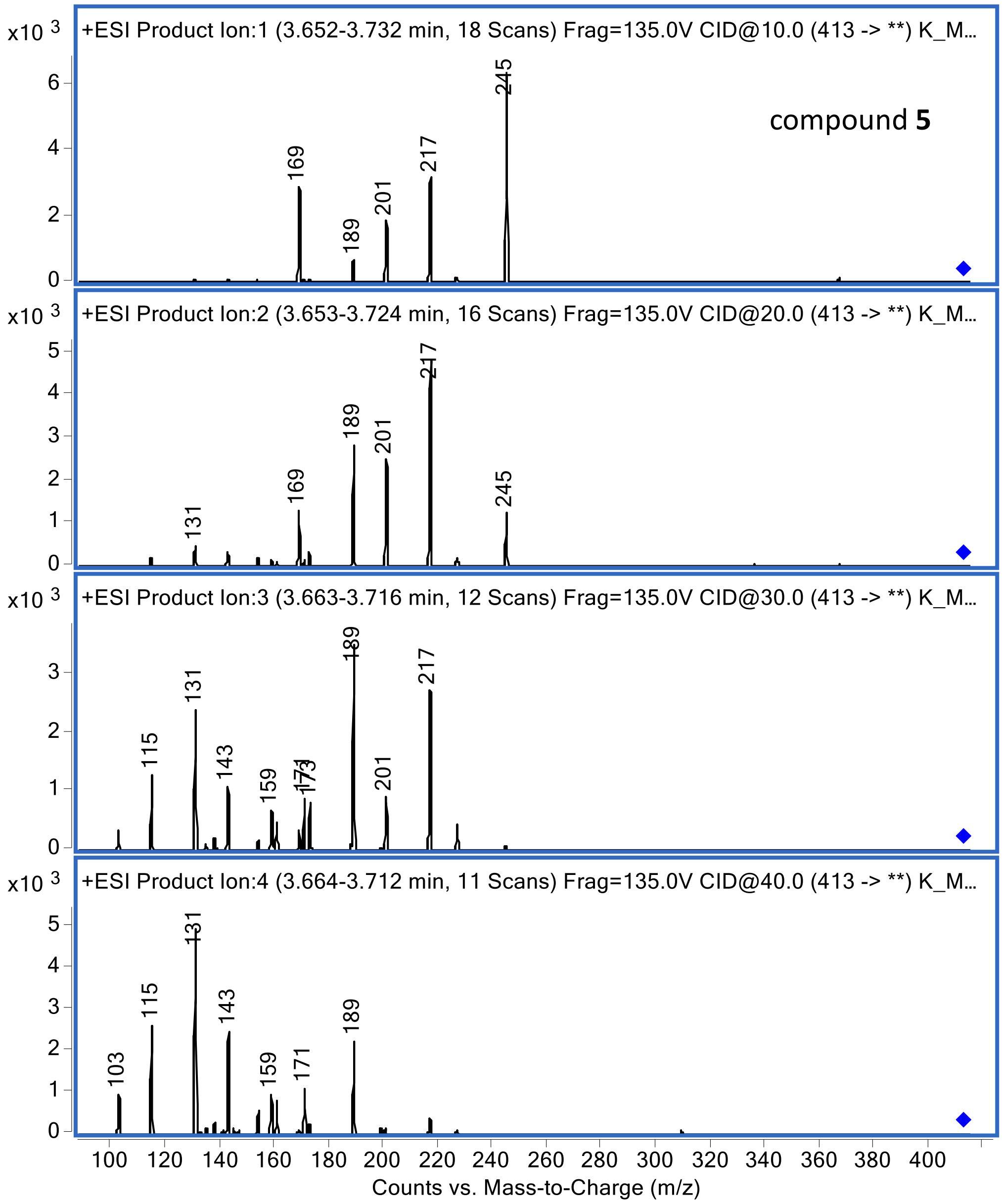

Figure S3. (continued)

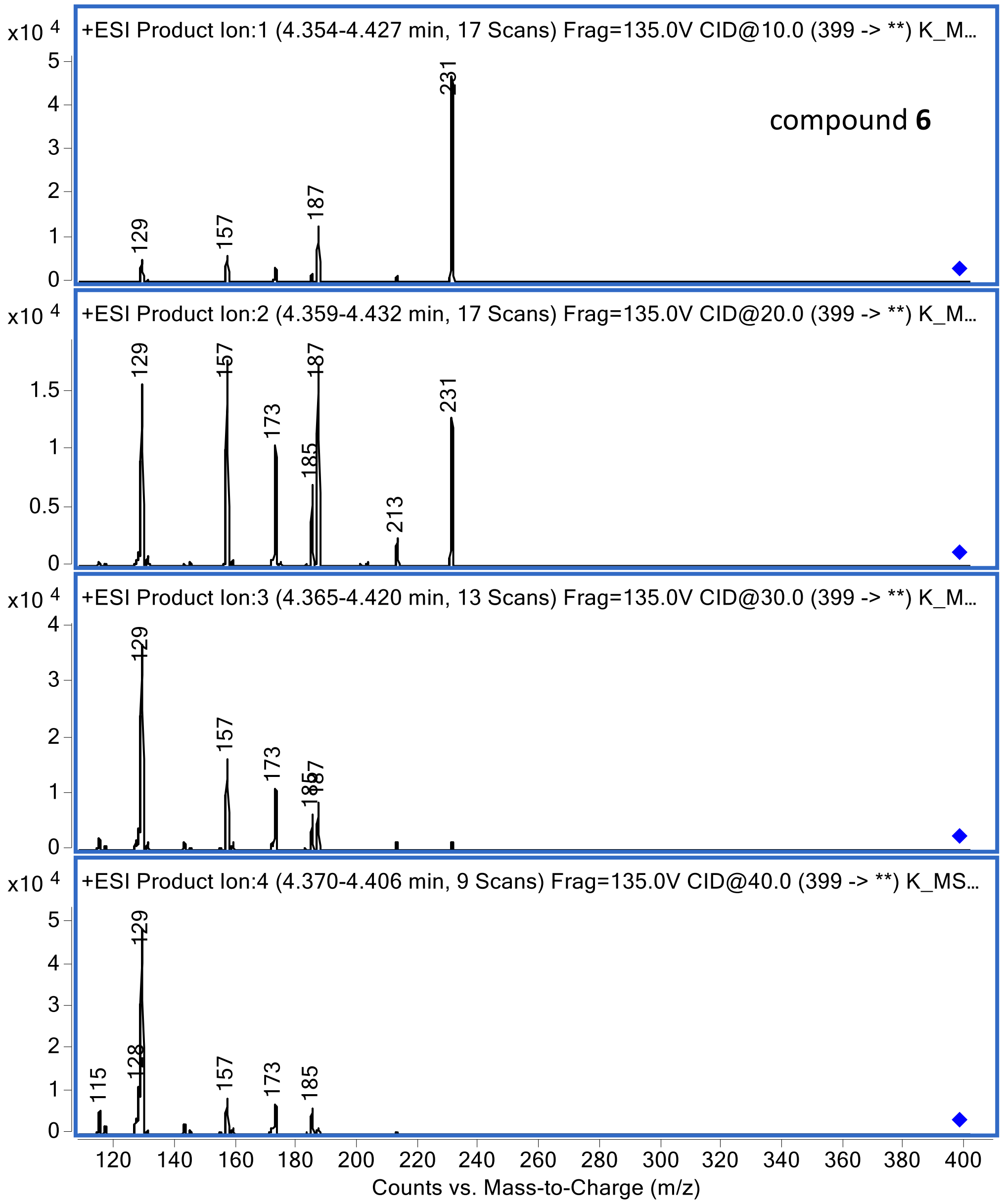

Figure S3. (continued)

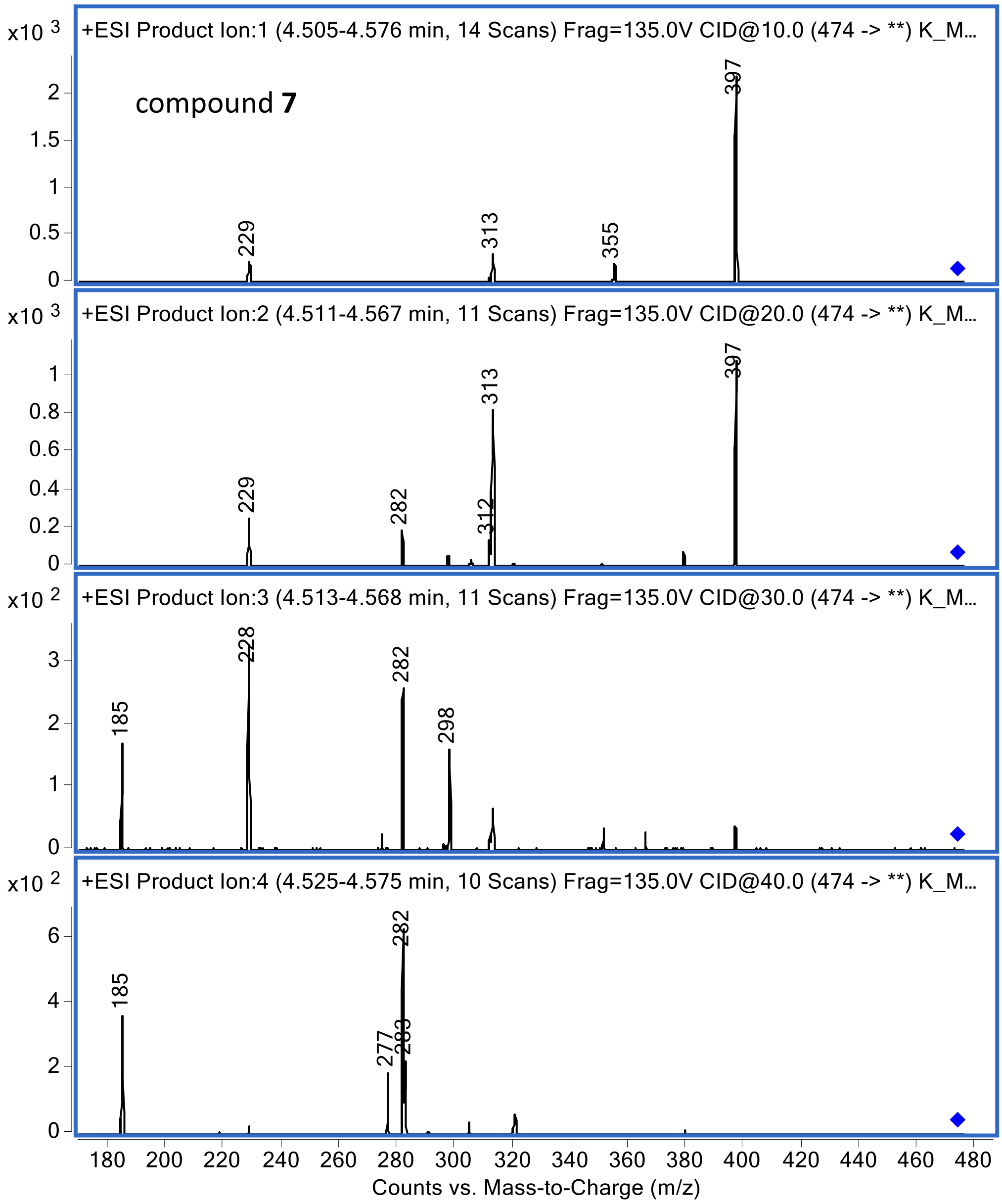

Figure S3. (continued)

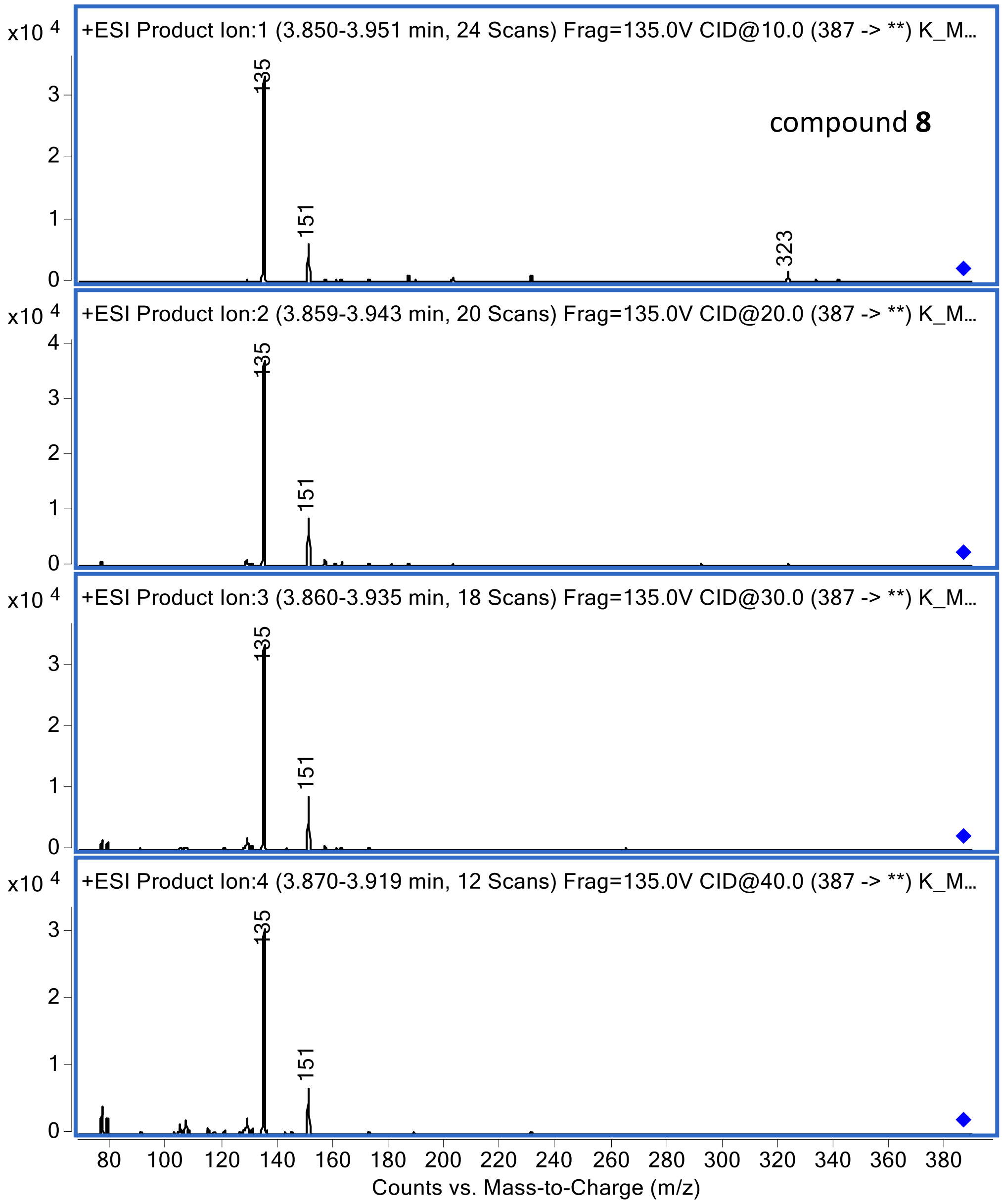

Figure S3. (continued)

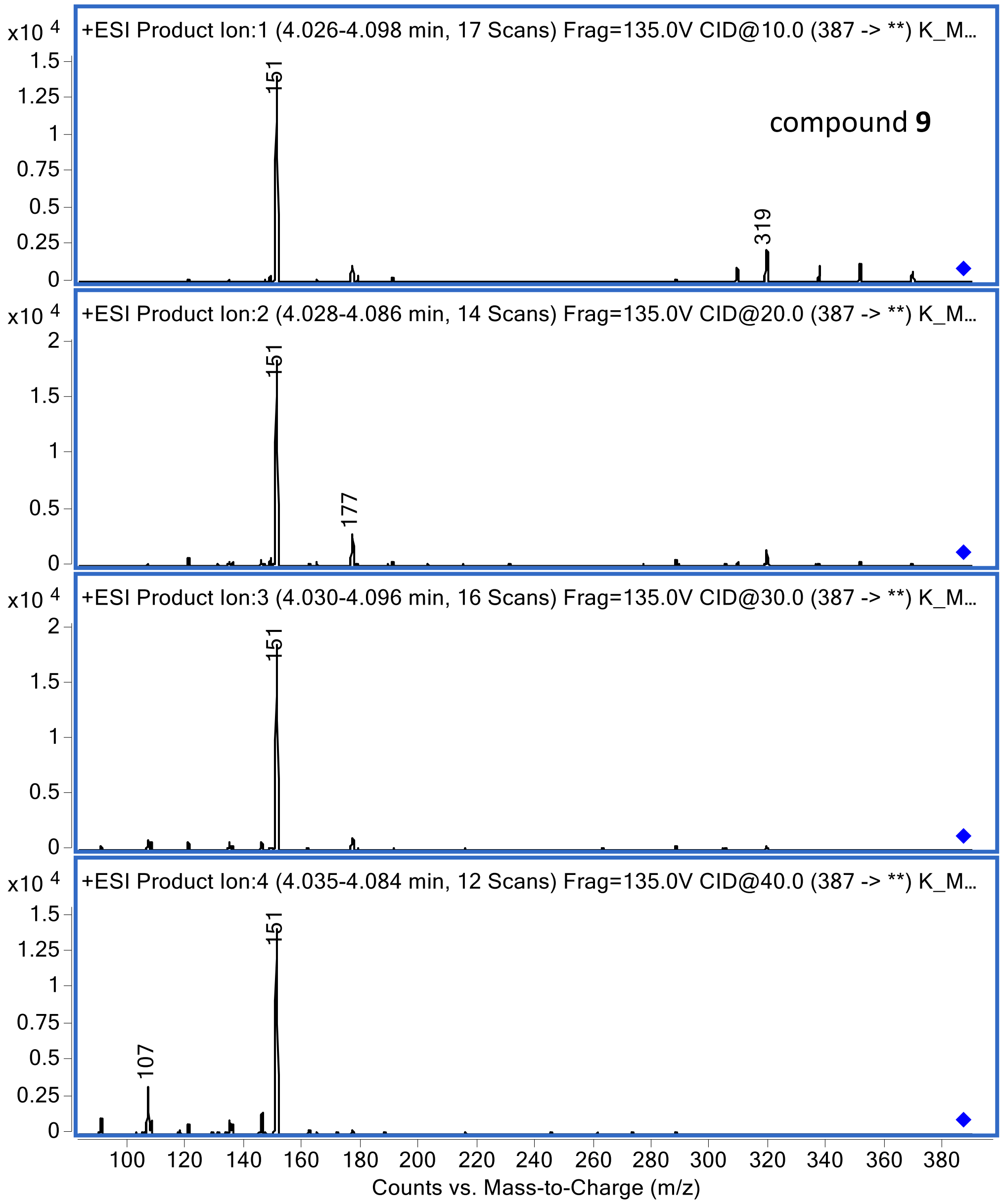

Figure S3. (continued)

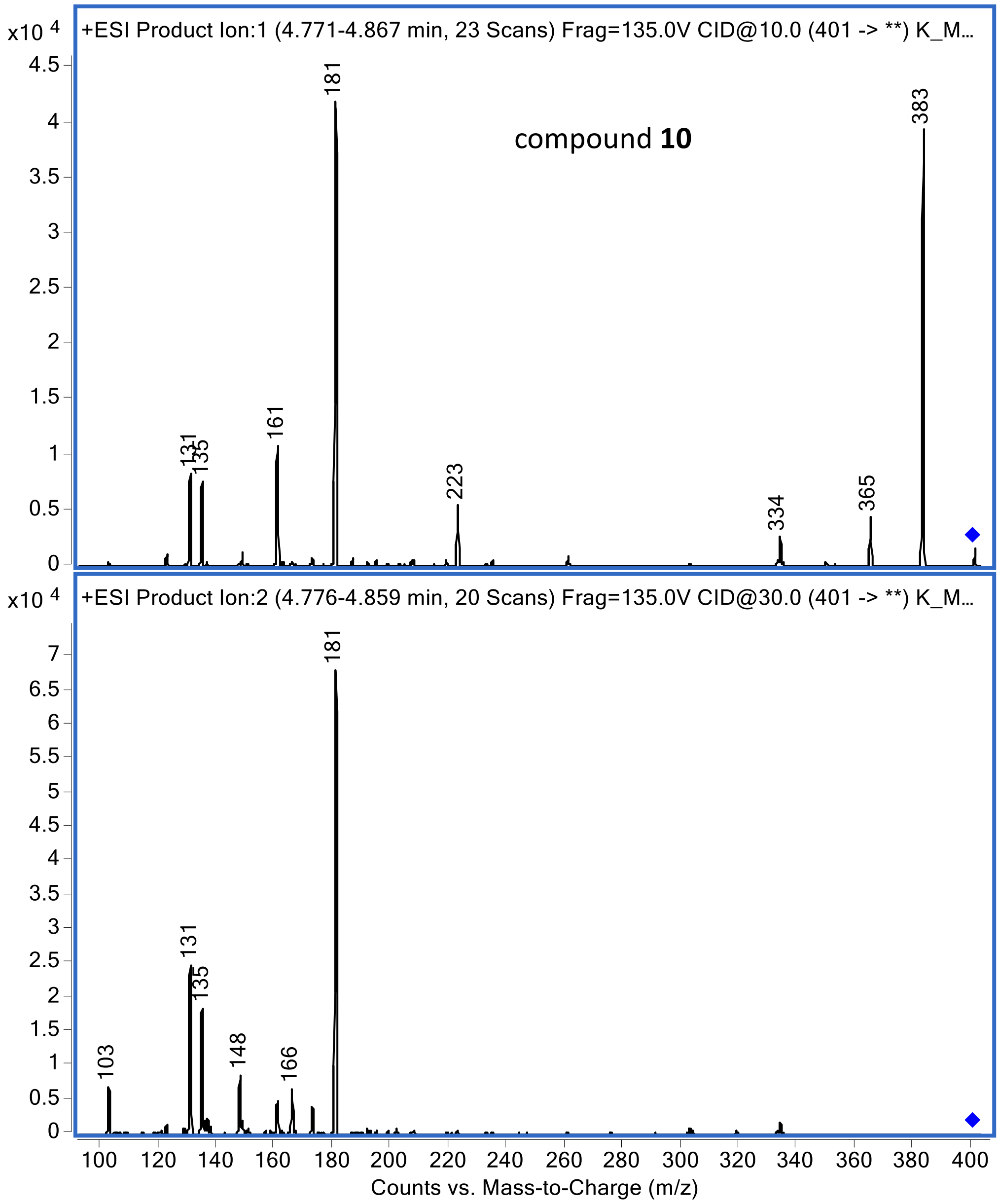

Figure S3. (continued)

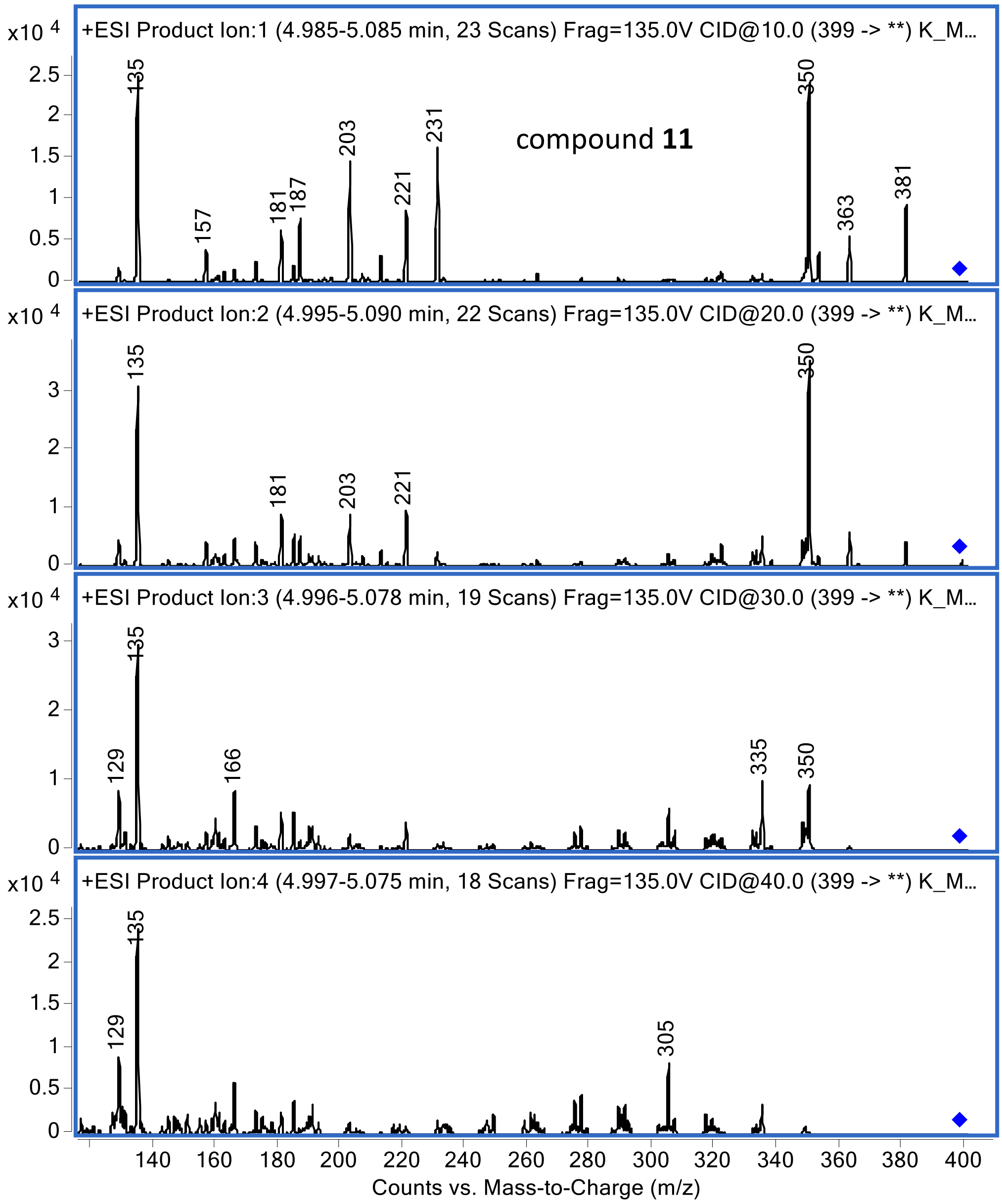

Figure S3. (continued)

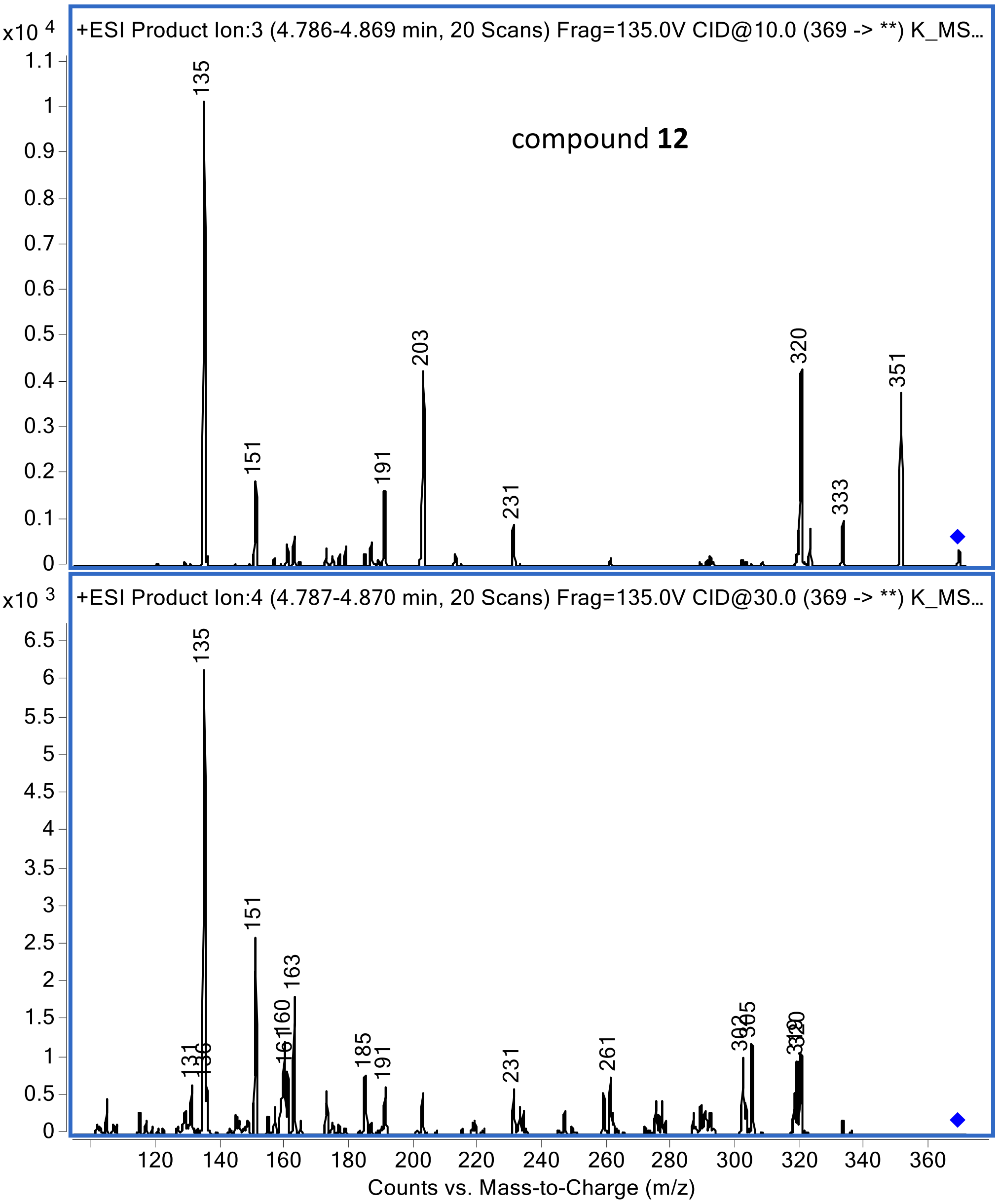

Figure S3. (continued)

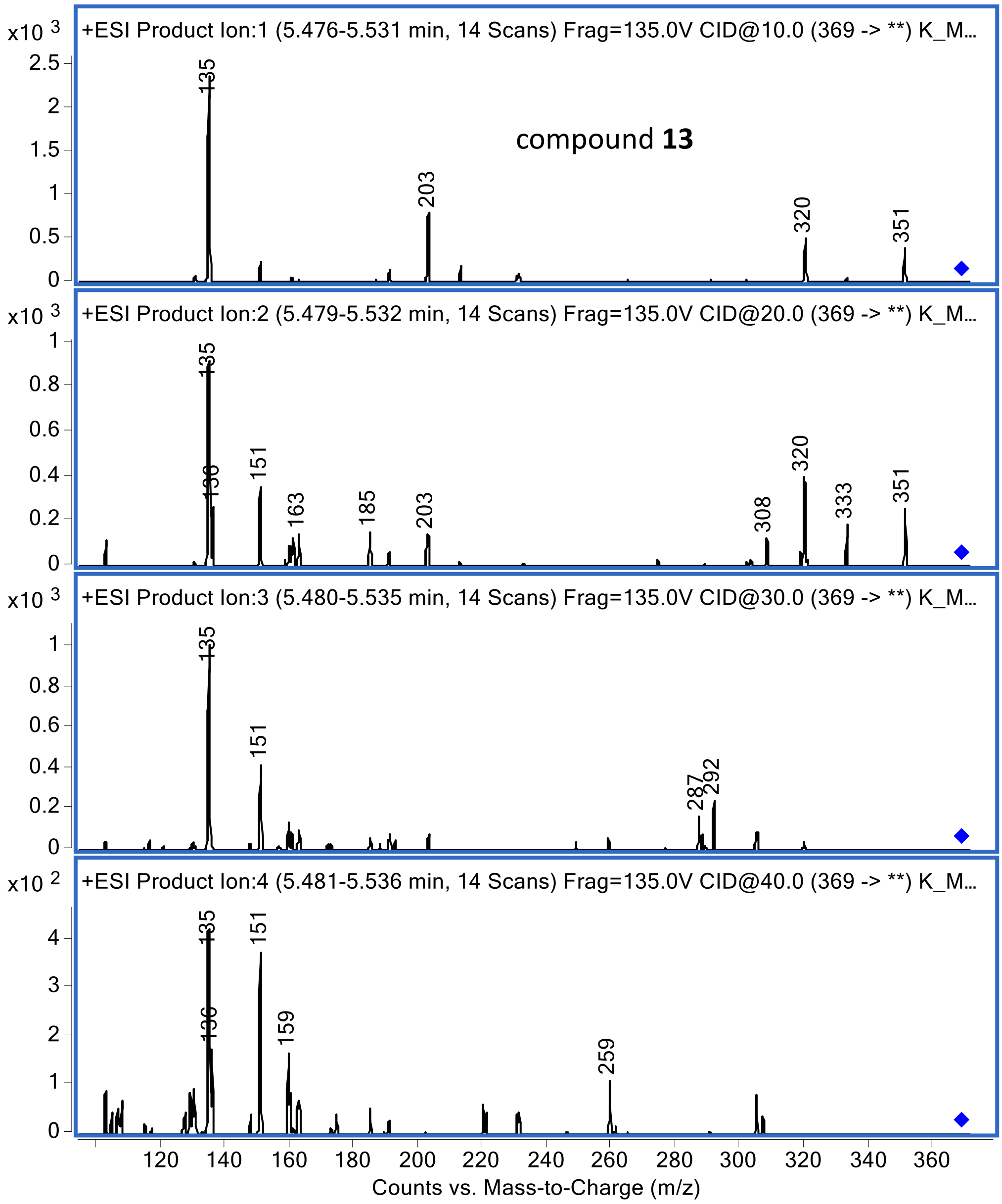

Figure S3. (continued)

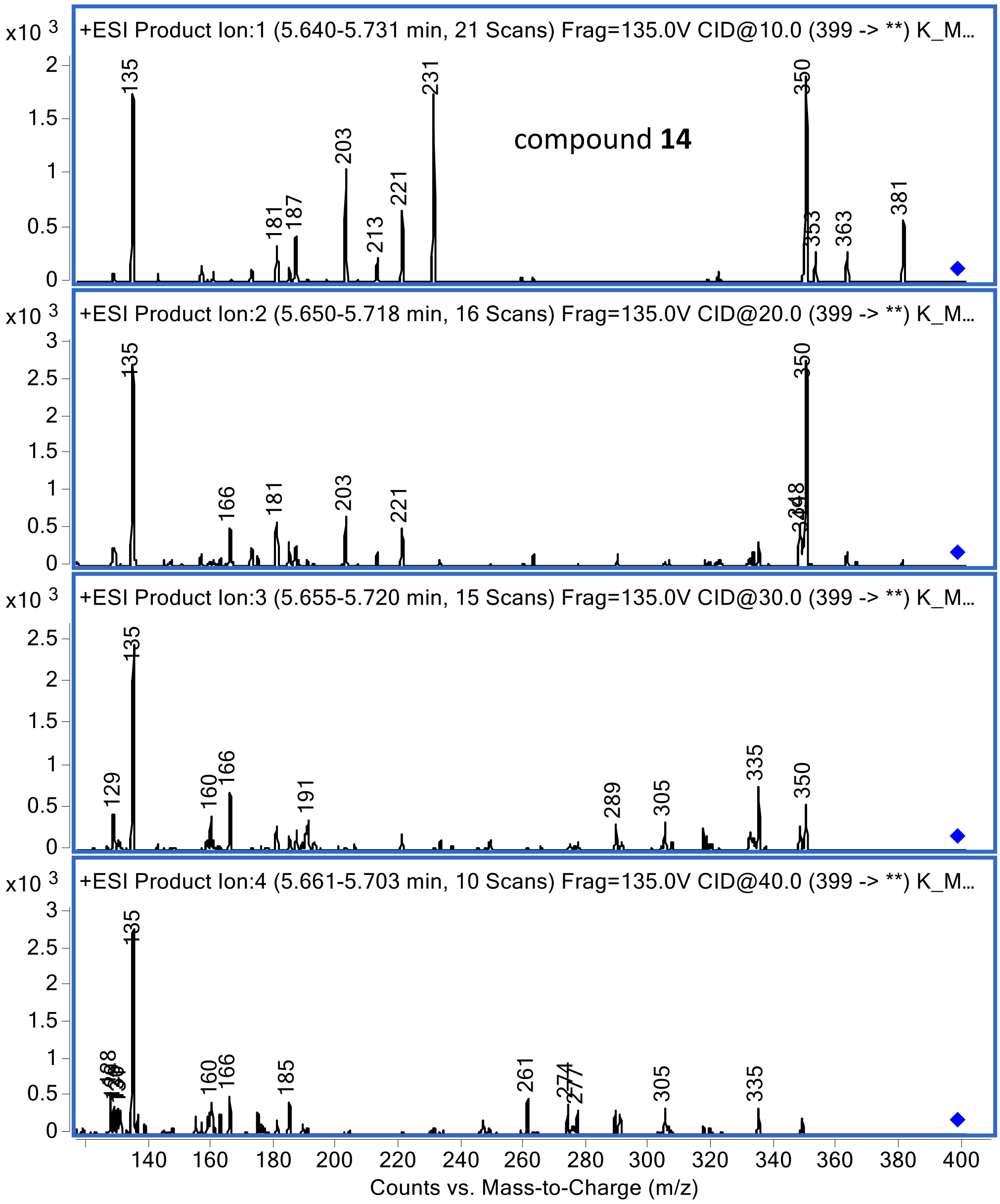

Figure S3. (continued)

Table S2. Linearity and detection ability parameters.

	linear range[a] /µg mL^{-1}	investigated range /µg mL^{-1}	regression eq.	r^2	LoD /µg mL^{-1}	LoQ /µg mL^{-1}
**1**	0.250-50.0	0.250-50.0	$y = 4.10680x$	0.99934	0.22	0.94
			$y = -0.0143458x^2 + 4.78858x$	0.99994		
**2**	2.50-250	0.312-250	$y = 1.52329x$	0.99982	1.5	2.5
**3**	0.125-10.0	0.125-10.0	$y = 14.72717x$	0.99938	0.043	0.10
**4**	0.25-10.0	0.125-50.0	$y = -0.0531437x^2 + 7.98000x$	0.99992	0.065	0.17
**5**	1.25-1000	1.25-1000	$y = 1.75542x$	0.99997	0.60	1.6
**6**	2.50-800	1.25-800	$y = 0.637284x$	0.99996	2.0	4.7
**7**	0.500-75.0	0.500-75.0	$y = 2.02948x$	0.99990	0.44	1.8
**8**	1.25-500	1.25-500	$y = 4.17542x$	0.99970	0.63	1.6
**9**	5.00-100	0.312-100	$y = 1.89180x$	0.99911	1.2	2.9
**10**	5.00-500	1.25-500	$y = 2.01727x$	0.99911	1.0	2.4
**11**	6.25-500	6.25-500	$y = 9.99418x$	0.99928	2.6	6.6
**12**	0.555-27.6	0.555-55.5	$y = -0.0706413x^2 + 15.466892x$	0.99904	0.31	0.87
			$y = 14.0323193x$	0.99918		
**13**	0.50-10.0	0.125-10.0	$y = 16.71686x$	0.99915	0.043	0.18
**14**	0.383-18.2	0.383-18.2	$y = 12.86720x$	0.99889	0.14	0.34

[a] defined as range resulting in satisfactory visual agreement, and relative residuals within 20 % (**SANCO**)

Table S3. Trueness and precision parameters.

	root		herb	
	recovery /%	repeatability /%	recovery /%	repeatability /%
**1**	78.8	7.2	92.1	3.7
**2**	76.6	8.6	100.3	5.1
**3**	79.2	5.8	86.9	3.0
**4**	83.6	4.7	106.9	6.6
**5**	76.5	4.3	63.0	8.7
**4+5**	73.1	4.3	78.1	7.2
**6**	95.1	4.5	88.1	6.2
**7**	97.5	7.1	104.1	3.3
**8**	86.8	6.1	89.5	2.5
**9**	86.9	5.7	89.0	3.2
**10**	91.2	4.5	85.0	6.7
**12+13**	-[a]	7.3	-[a]	13.5
**11+14**	99.4	4.5	88.1	6.7

[a] not determined due to insufficient amount of standard available

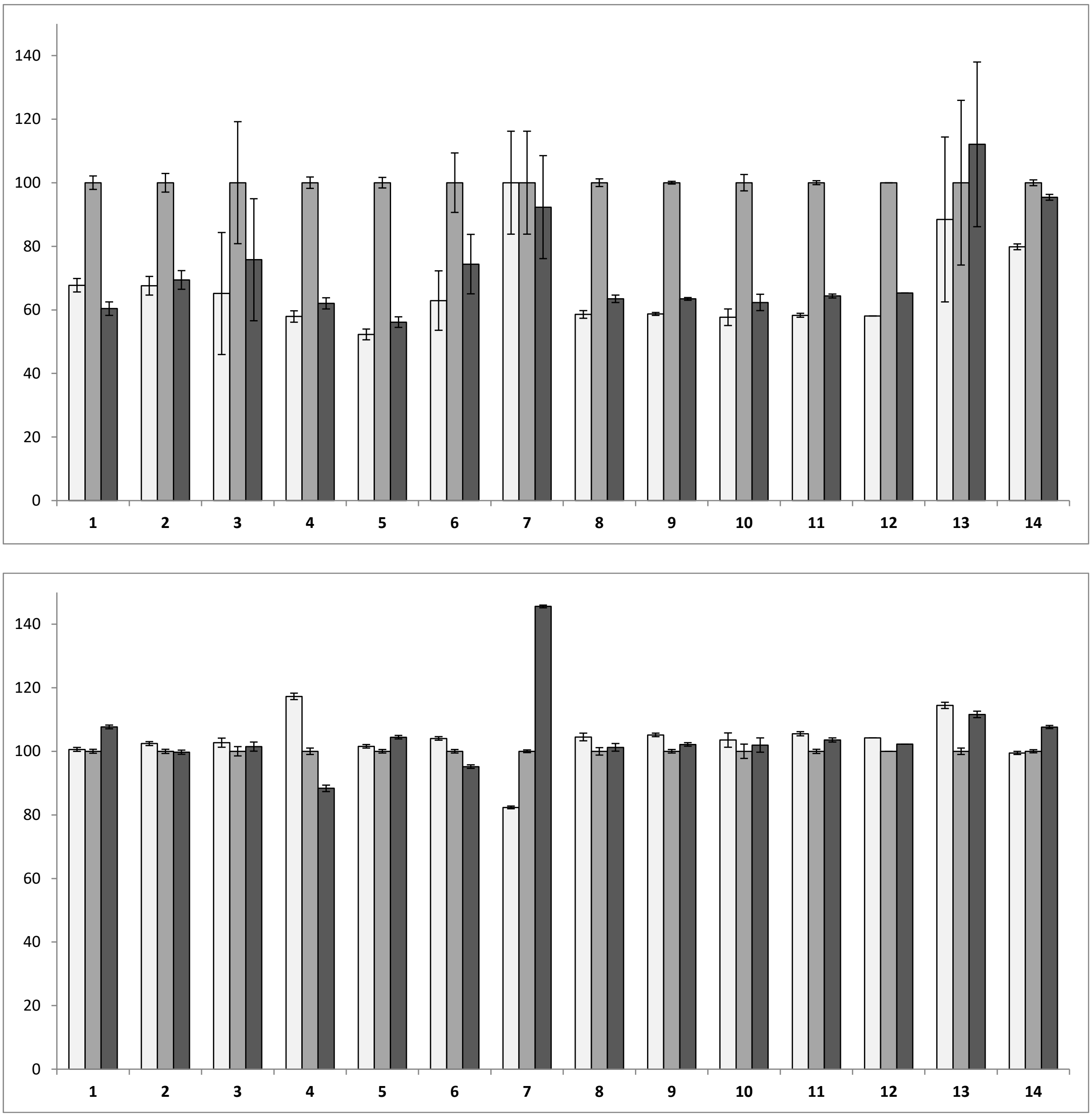

Figure S4. The effect of the extraction solvent composition (70 %, 80 % and 90 % MeOH) on the extraction yield (given as percentage of yield with 80 % MeOH) from aerial parts (top) and roots (bottom).

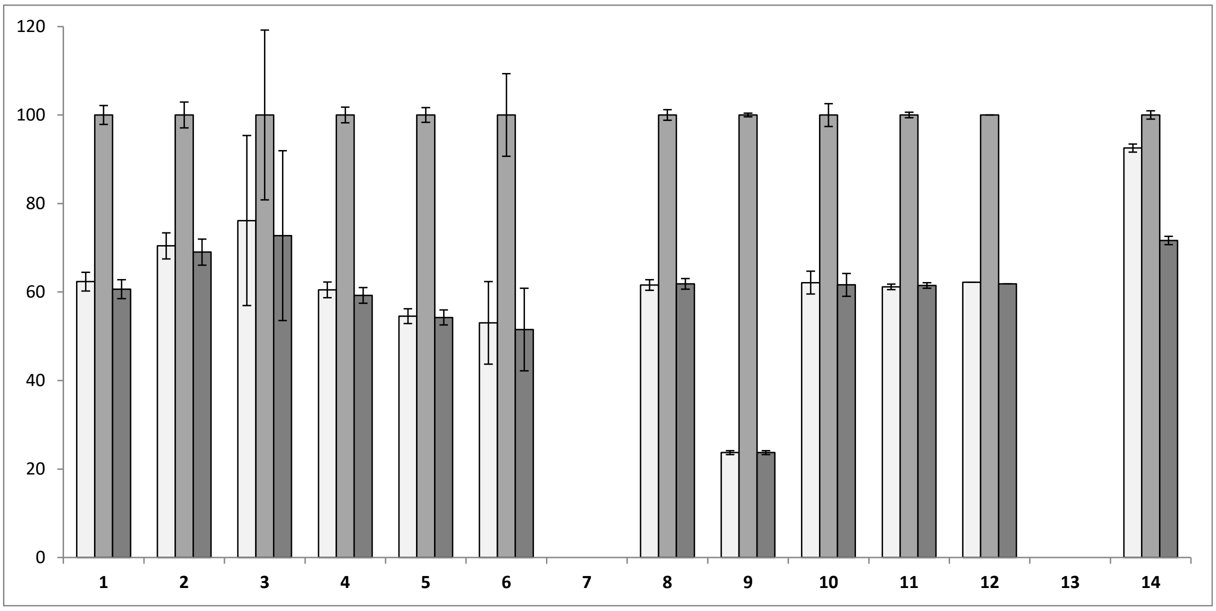

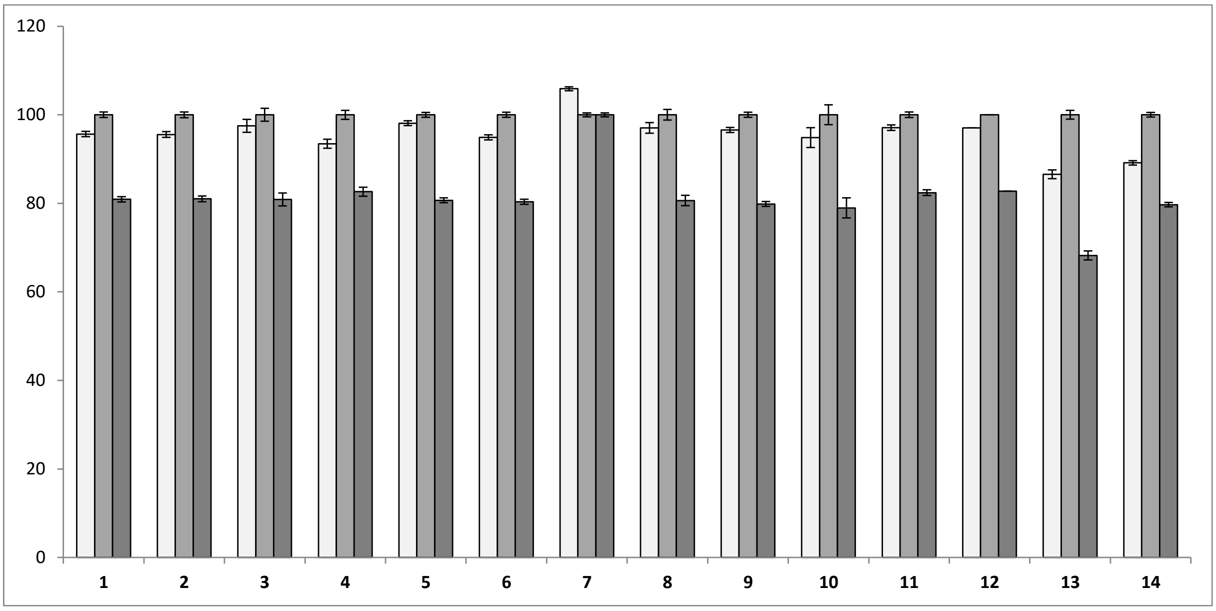

Figure S5. The effect of the extraction time (45 min, 60 min, 75 min) on the extraction yield (given as percentage of yield at 60 min) from aerial parts (top) and roots (bottom).

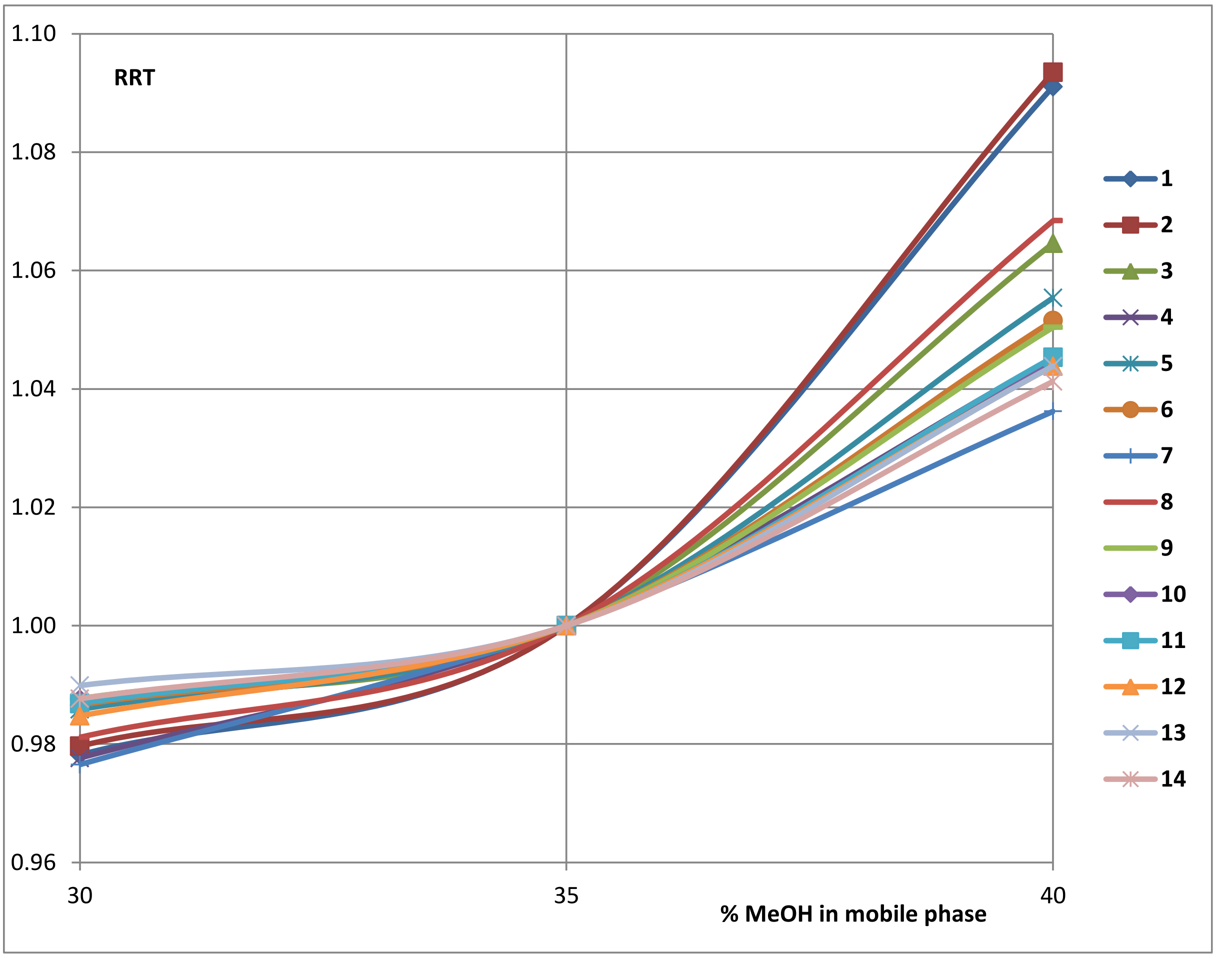

Figure S6. The effect of MeOH content in the mobile phase (30 %, 35 %, 40 %) on the retention time (given as relative retention times, RRT, against the retention times obtained with 35 % MeOH).

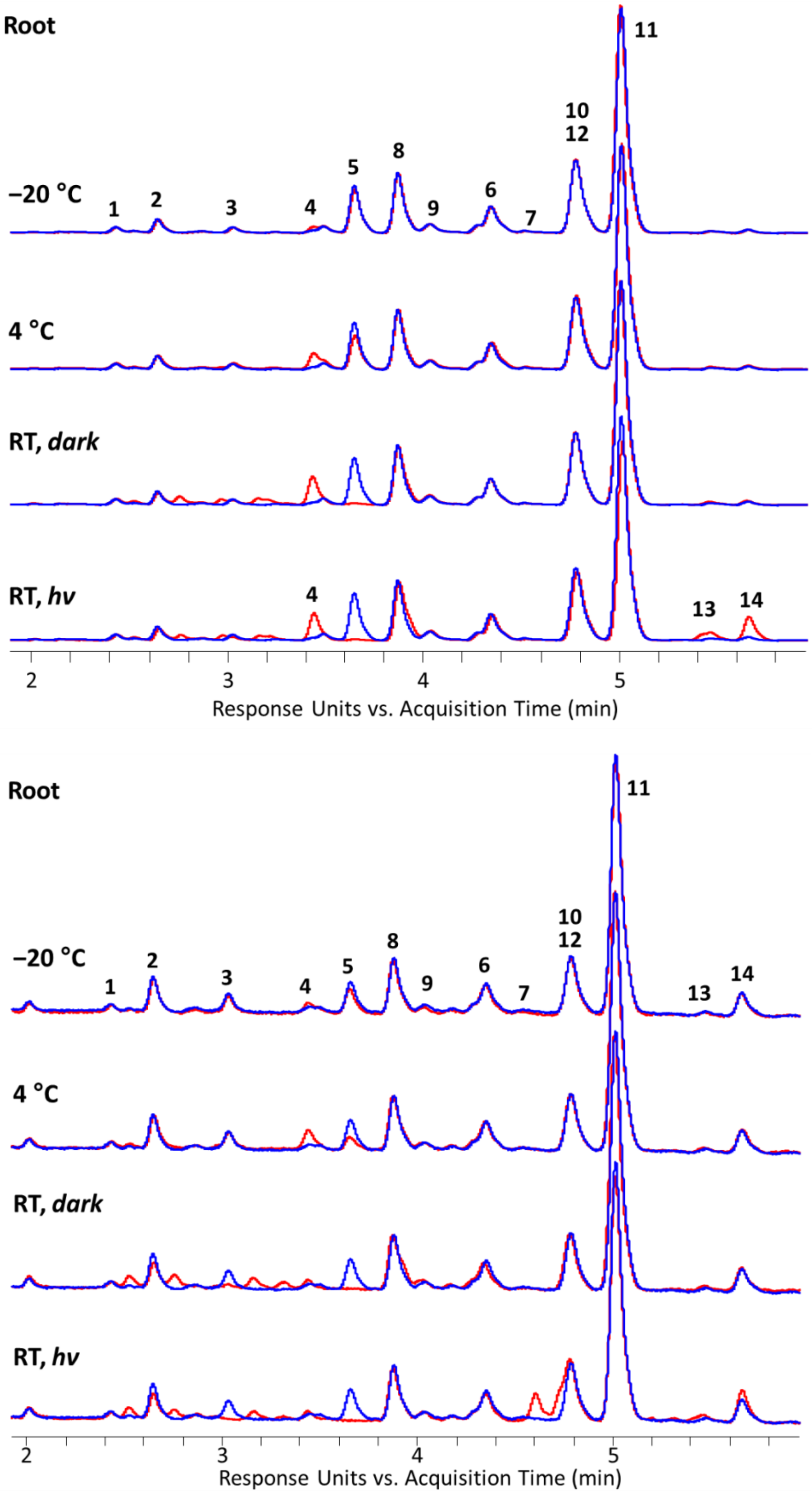

Figure S7. Stability evaluation after four weeks of storage: 280 nm chromatograms of sample stored at –80 °C (blue) and at –20 °C, 4 °C, and room temperature in dark and under normal illumination (red).

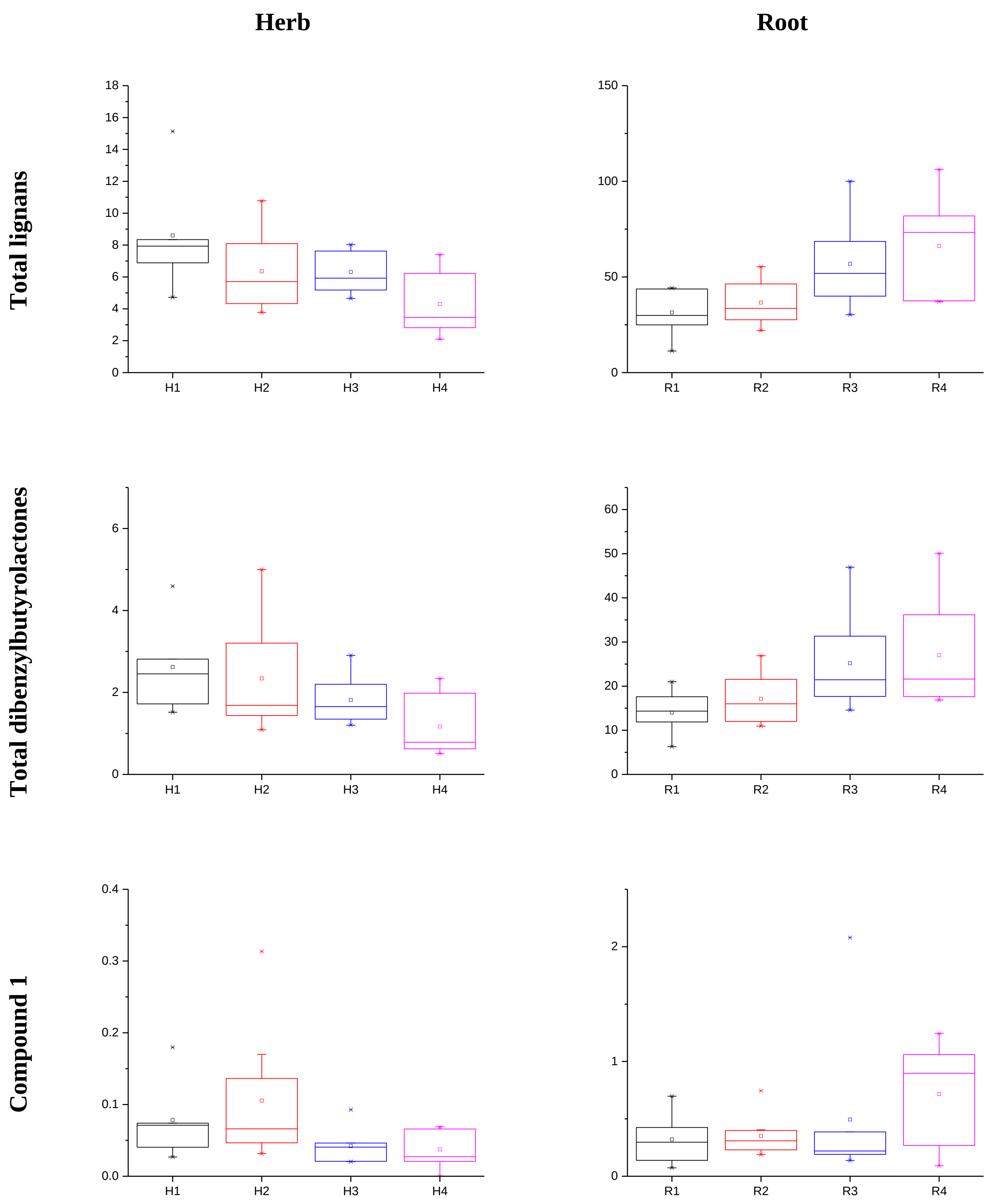

Figure S8. Changes in lignans content (in mg/g d.w.) during the plant development. Quartiles Q_1 and Q_3 are denoted by box boundaries, median by box-dividing line, mean by a quare point, range (Q_1–1.5 IQR, Q_3+1.5 IQR) by whiskers, and outliers by cross points.

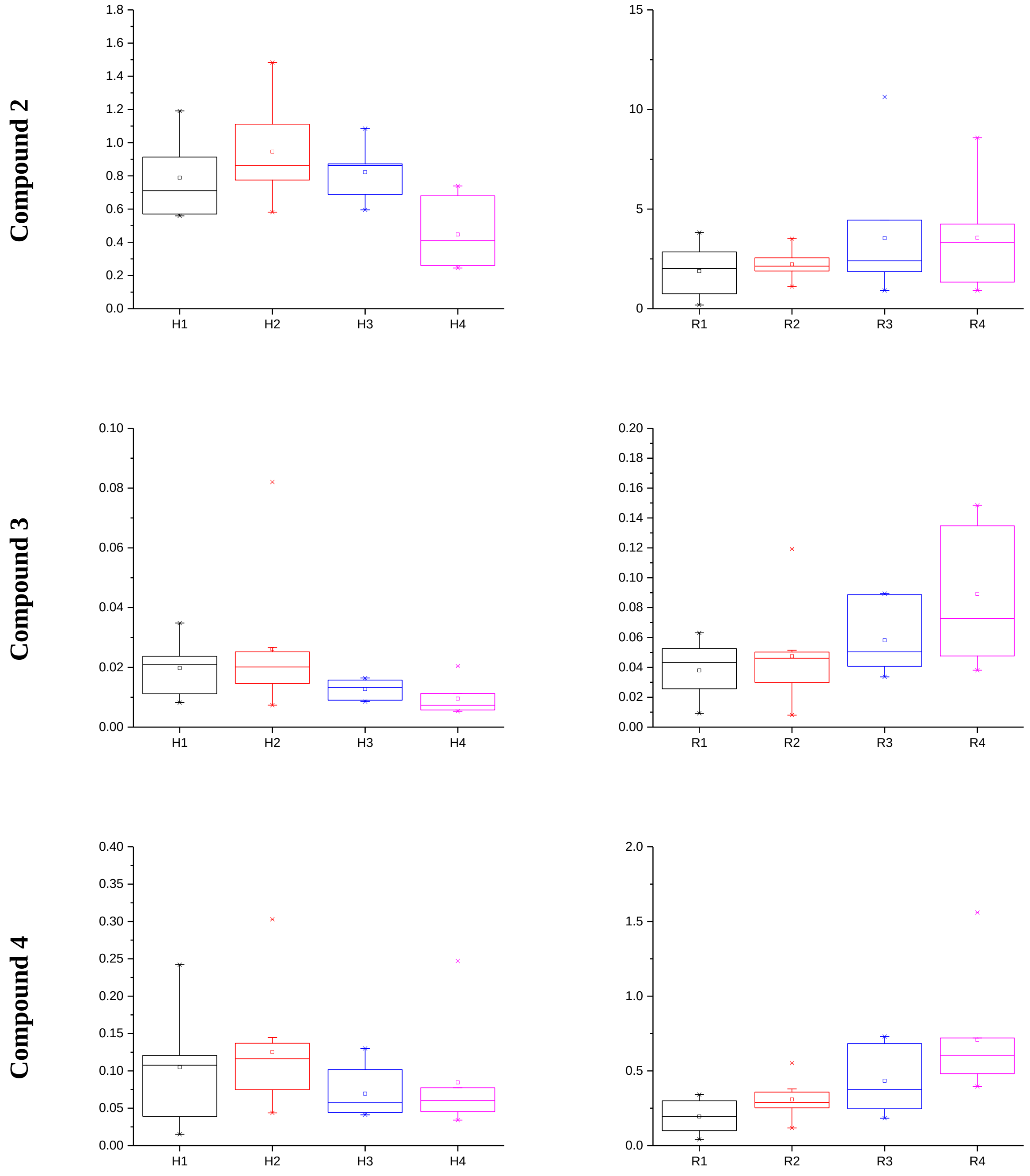

Figure S8. (continued)

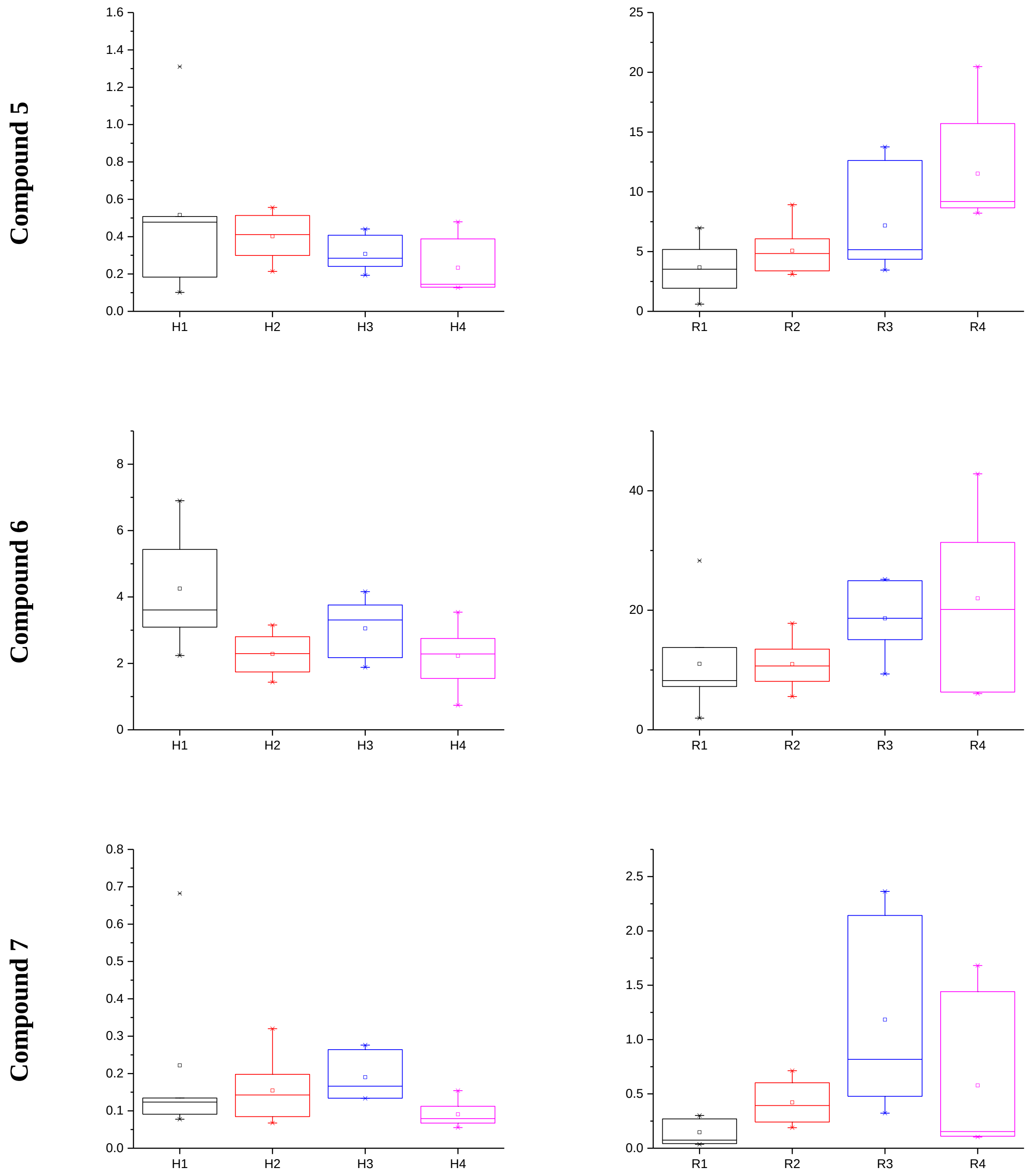

Figure S8. (continued)

Compound 8

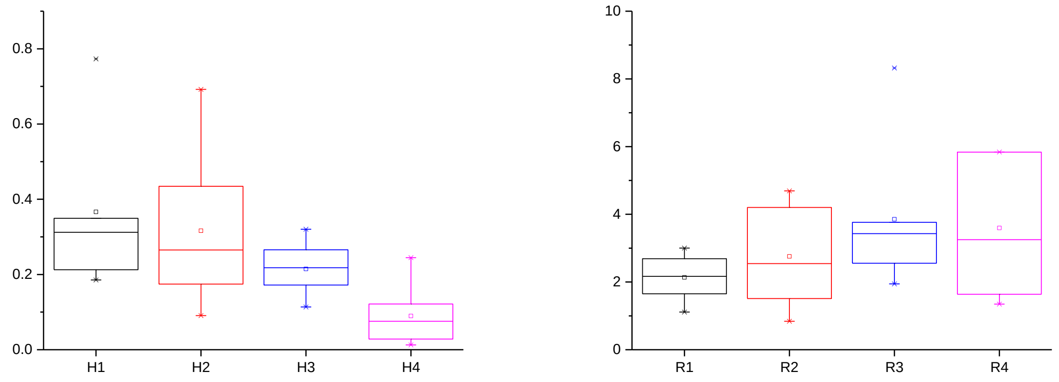

Compound 9

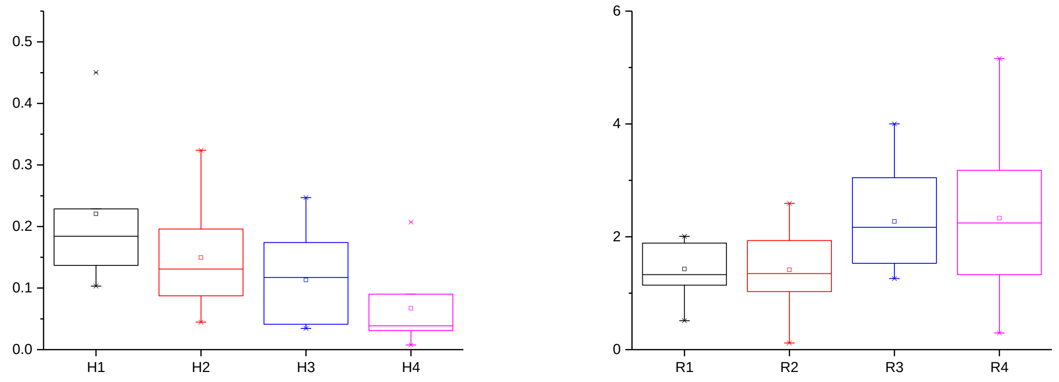

Compound 10

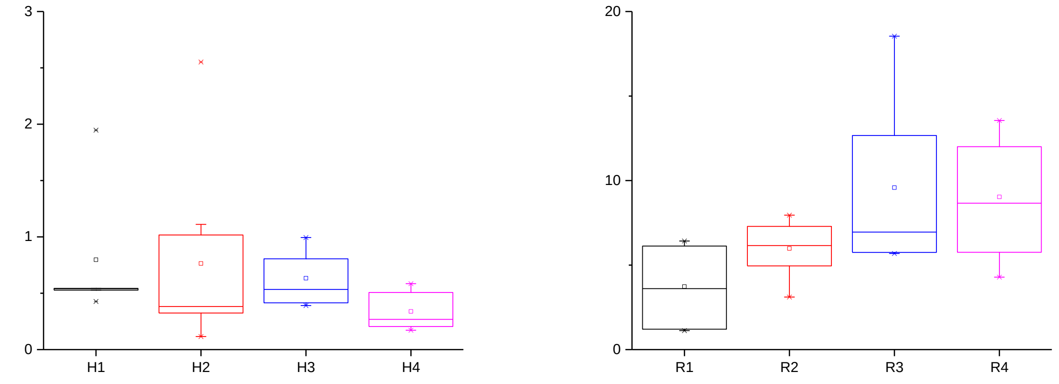

Figure S8. (continued)

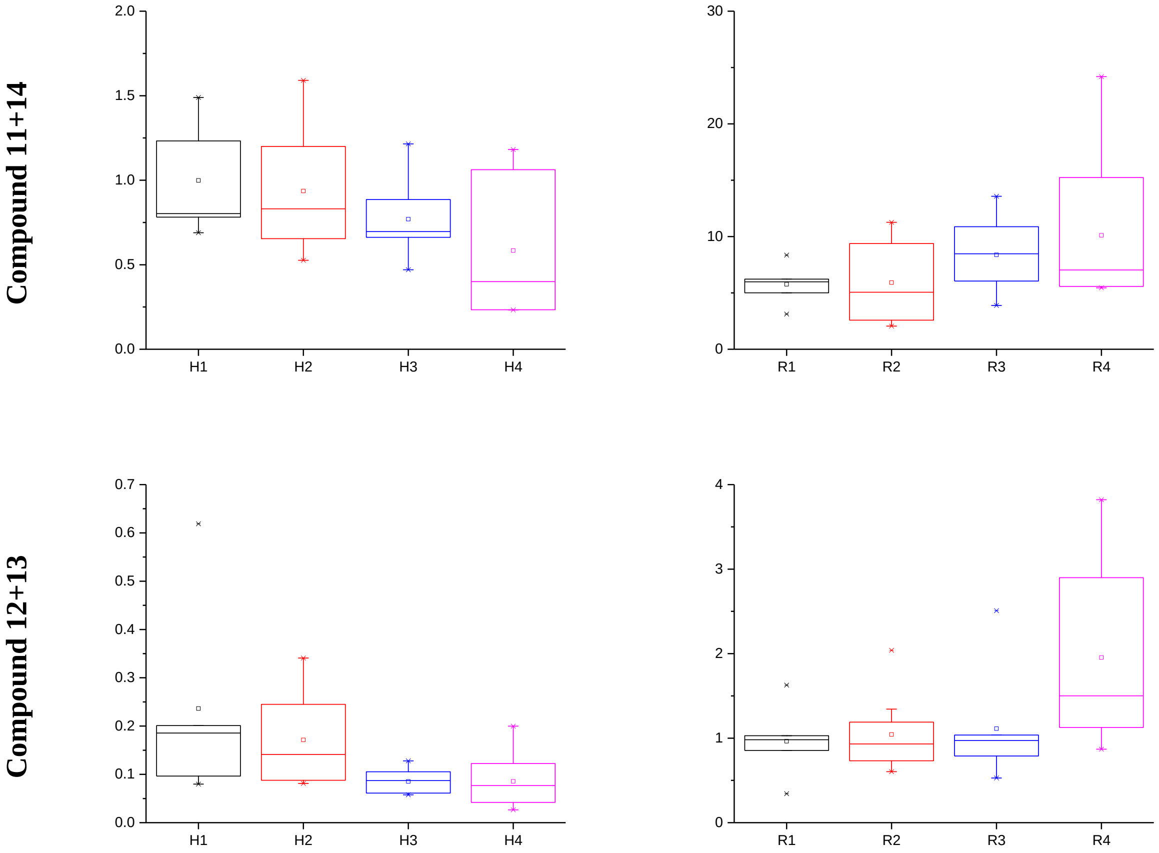

Figure S8. (continued)
